# Lysine lactylation in diseases: beyond histone lactylation

**DOI:** 10.1038/s41419-025-08223-6

**Published:** 2025-11-24

**Authors:** Yiming Liu, Xuan Guo, Xinyang Hu, Sining Zhou, Qi Yang, Pingping Feng, Linghui Zeng

**Affiliations:** 1 Hangzhou Lin’an Traditional Chinese Medicine Hospital, Affiliated Hospital, Hangzhou City University, Hangzhou, 311300 China; 2 Laboratory of Cancer Biology, Key Laboratory of Biotherapy of Zhejiang Province, Sir Run Run Shaw Hospital, Zhejiang University School of Medicine, Hangzhou, 310017 China; 3 Key Laboratory of Novel Targets and Drug Study for Neural Repair of Zhejiang Province, Hangzhou City University School of Medicine, Hangzhou, 310015 China; 4 Cancer Center, Zhejiang University, Hangzhou, 310058 China; 5 Life Sciences Institute, Zhejiang University, Hangzhou, 310058 China

**Keywords:** Cancer, Cardiovascular diseases, Neurological disorders, Infectious diseases, Immunological disorders

## Abstract

Lactylation, a recently identified post-translational modification, was initially characterized as lysine residue modification in histone subunits that regulates gene transcription via epigenetic mechanisms. Elevated intracellular lactate has been shown to drive histone lysine lactylation (Kla), establishing its association with disease pathogenesis. Emerging evidence reveals that Kla modifications extend beyond histones to transcriptional regulators and cytoplasmic functional proteins. Unlike the broad transcriptional regulation mediated by histone lactylation, Kla modifications of functional proteins exert regulatory effects through both specific transcriptional modulation and direct functional alteration of target proteins, thereby precisely controlling biological processes. This review systematically examines the pathological implications of Kla modifications of functional proteins across multiple disease contexts, including inflammatory disorders, infectious diseases, neurological or cardiovascular pathologies, and oncological conditions. Our synthesis provides mechanistic insights into disease-associated Kla networks, facilitating therapeutic target discovery and pharmacological intervention strategies.

## Facts

- Kla modifications on non-histone proteins, such as transcription factors and enzymes, exhibit regulatory effects on gene transcription and protein function, but the precise mechanisms distinguishing pathological from physiological Kla modifications remain unclear, warranting further investigation into context-specific writers and erasers.
- While elevated lactate-driven Kla modifications have been linked to disease progression in inflammation, infections, neurological disorders, cardiovascular pathologies and cancers, the therapeutic potential of targeting specific Kla-modified proteins for pharmacological intervention is underexplored and could reveal novel druggable pathways.
- The crosstalk between Kla modifications and other PTMs on functional proteins may amplify or antagonize disease phenotypes, posing a key debatable area for future studies on integrated PTM networks in pathological conditions.
- Emerging evidence suggests Kla modifications extend to cytoplasmic and organellar proteins beyond the nucleus, but their roles in metabolic reprogramming and cellular homeostasis during disease states require further mechanistic elucidation to guide precision medicine approaches.

## Introduction

Enhanced glycolytic flux, a hallmark of diverse pathological states including inflammation, organ injury, viral infections, neurological damage, cardiovascular disorders, and malignancies, drives lactate dehydrogenase A (LDHA) upregulation and consequent lactate overproduction [1–6]. In 2019, pioneering work from the University of Chicago employed liquid chromatography-tandem mass spectrometry (LC-MS/MS) to identify lysine lactylation (Kla) sites on histone H3 across human and murine cells [7]. This breakthrough revealed that Kla modification on K18 of histone H3 (H3K18la) functions as a transcriptional activator. These findings established Kla as a pivotal player in post-translational regulation, sparking intense research interest in its epigenetic roles.

The eukaryotic nucleosome core comprises an octamer of histone subunits (H2A, H2B, H3, H4) that organizes genomic DNA [8, 9]. Dynamic post-translational modifications (PTMs) such as methylation and acetylation at specific histone residues modulate histone-DNA interactions, thereby regulating chromatin compaction states and transcriptional accessibility [10–13]. Emerging evidence now extends this paradigm to lactylation, with the Kla-modified sites identified on H3, H4, and H2B subunits, though H2A remains conspicuously devoid of detectable Kla modification [14–16]. Among Kla-modified histones, histone H3 exhibits the highest lactylation density and functional significance, particularly at the H3K18 site [17–19].

Elevated H3K18la levels can activate inflammation-related pathways, thereby promoting oxidative stress and apoptosis, which ultimately lead to renal dysfunction [20]. Similarly, in multiple cancers, increased H3K18la levels can regulate the transcription of genes associated with tumor growth and metastasis, thereby facilitating tumorigenesis and progression [19, 21, 22]. Besides, intracellular lactate stress can also activate Kla modifications of histone H3 at K9, K14, and K56 to remodel chromatin structure and facilitate gene transcription, further promoting inflammation and cancer progression [17, 23–25].

Kla modifications on histone H4 have also been shown to play significant roles in regulating gene transcription. Specifically, the H4K12la modification is considered to be critical in the onset and development of neurological, oncological, and cardiovascular diseases [15, 26–28]. H4K12la can activate the expression of inflammation and glycolysis-related proteins, thereby promoting the progression of Alzheimer’s disease through the activation of microglial autophagy-related signaling pathways and glycolysis [26, 27]. Moreover, H4K12la can also facilitate the progression of atherosclerosis and tumors by activating the transcription of genes associated with aging and metabolism [15, 28]. Additionally, H4K5la and H4K8la have also been reported to promote disease progression [29–31].

In contrast, modifications of histone H2B have been less frequently reported. In hepatocellular carcinoma (HCC), histone H2B can be Kla-modified at K58, thus facilitating the transcription of *NDRG1* to inhibit cell senescence and apoptosis [16]. Moreover, H2BK16la modification has been identified in cells infected by classical swine fever virus (CSFV), which mediates the nuclear translocation of p65 via karyopherin α2 (KPNA2), thereby inducing the expression of interferon-λ (IFN-λ) to counteract viral infections [32].

Overall, Kla modifications on histone subunits exhibit extensive regulatory functions in gene transcription, playing a pivotal role in both the pathogenesis of diseases and the maintenance of normal physiological functions.

Some studies have used immunofluorescence staining to detect total cellular Kla modification, and it is clear that Kla-modified proteins are predominantly distributed in the nucleus, although signals are also present in the cytoplasm and some organelles [33, 34]. Immunoblotting analysis of whole cell lysates has revealed that although histone H3 and H4 show the strongest signals of Kla modification, other proteins also exhibit Kla modifications [35, 36]. These studies indicate that Kla modification occurs not only on histones but also on other functional proteins, thus regulating cellular physiological and biochemical functions.

The following studies have shown that Kla modification is present on transcription factors (TFs), enzymes, secretory proteins, and cellular signaling-related proteins [20, 37–39]. Kla modification regulates the activation and nuclear translocation of TFs, protein secretion, the interactions between signaling-related proteins and their downstream proteins, the activity of enzymes, including metabolic enzymes, E3 ubiquitin ligases, and mRNA modification-related enzymes [20, 39–43]. Additionally, some studies have also demonstrated that Kla modification not only impacts the function of proteins but also regulates protein stability, thereby upregulating specific proteins and influencing downstream physiological and biochemical functions [44, 45].

In this review, we have summarized the recent studies on Kla modifications of functional proteins according to the research fields, in order to provide direction for future studies on Kla modification.

## The Kla modifications of functional proteins in inflammation

**Fig. 1:**
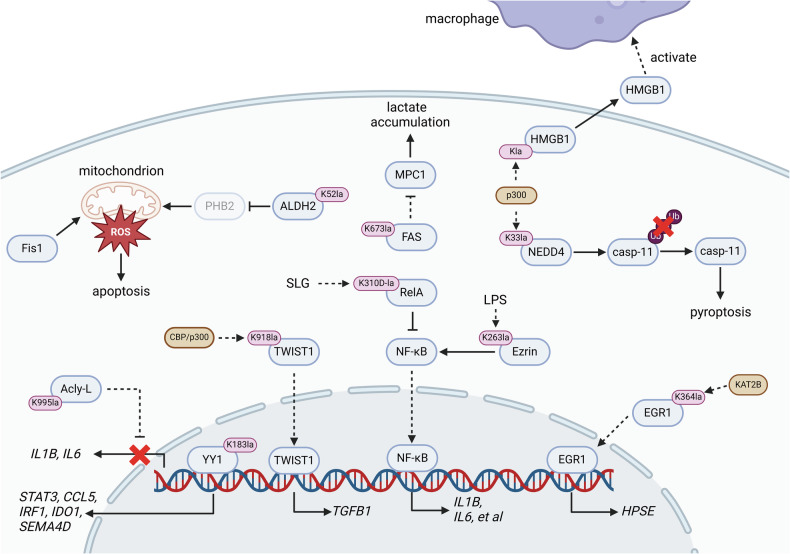
The Kla modifications of functional proteins in inflammation. The metabolic reprogramming of immune cells leads to elevated lactate levels within cells during acute and chronic inflammation, leading the Kla modifications of functional proteins to facilitate or inhibit the process of inflammation. The Kla modifications of Acly-L, YY1, TWIST1, Ezrin, RelA and EGR1 facilitate or inhibit the transactivation of pro-inflammatory cytokines to aggravate or relieve inflammation. Besides, the Kla-modified ALDH2 and NEDD4 could also facilitate inflammation via inducing cell apoptosis or pyroptosis. The Kla-modified FAS can reduce the level of MPC1, resulting in accumulation of intracellular lactate to facilitate the process of glycolysis. Moreover, the Kla-modified HMGB1 can be secreted into the extracellular spaces to activate the chemotaxis of macrophages, causing cell damage.

Inflammation is an immune response produced by the body to damaged tissues and external stimuli [46]. It is a defense mechanism aimed at isolating and eliminating the source of tissue damage, as well as repairing the tissues [47, 48]. In inflammatory tissues, local macrophages, dendritic cells, and T lymphocytes are activated to release various cytokines and chemicals such as interleukins (ILs), tumor necrosis factors (TNFs), granzyme B, and histamine [49–51]. These molecules exert functional roles in killing pathogen-infected or necrotic cells, promoting vasodilation and increasing vascular permeability [52–54]. However, if the stimulus is not promptly removed and continues to affect these cells, it may lead to excessive secretion of these cytokines and chemicals [55]. This process can activate pro-inflammatory cytokines, such as IL-1β and IL-18, exacerbating tissue inflammation, leading to cellular damage, and ultimately resulting in inflammatory diseases [56, 57]. Many studies have confirmed that inflammation promotes glycolysis, leading to enhanced LDHA activity and lactate accumulation in the microenvironment [58–61]. Therefore, the studies of Kla modifications of functional proteins in inflammatory diseases are actively underway. Herein, we will discuss the specific roles of Kla modifications in modulating inflammatory responses (Fig. 1).

Cytokine storm, primarily caused by the transcriptional activation of pro-inflammatory cytokines, is considered as the dominant factor contributing to inflammatory diseases [62, 63]. The excessively released pro-inflammatory cytokines can cause vascular damage and organ failure through activating cell death [64, 65]. Some studies have indicated that Kla modifications promote the nuclear translocation of EGR1 and TWIST1, thus activating the transcription of genes encoding heparinase or pro-inflammatory cytokines, resulting in lung injury and skin fibrosis [37, 66]. In addition to promoting the nuclear entry of TFs, Kla modification can also regulate the activity of the TFs themselves, thereby modulating the transcriptional activation of pro-inflammatory cytokines. In autoimmune uveitis, the elevated lactate levels in microglia can lead to the Kla modification of Yin Yang 1 (YY1), thereby promoting the transcriptional activation of genes such as signal transducer and activator of transcription 3 (STAT3), chemokine (C-C motif) ligand 5 (CCL5), indoleamine 2,3-dioxygenase 1 (IDO1), and semaphorin 4 A (SEMA4A), which in turn promotes the development of inflammation [67].

The high lactate environment, in addition to regulating TFs through Kla modifications, can also promote the development of inflammatory diseases by modulating enzymatic activities. In acute kidney injury, ALDH2 is Kla modified at K52, which exacerbates tubular damage and mitochondrial dysfunction via attenuating its interaction with prohibitin 2 (PHB2), thus facilitating the ubiquitin-proteasome-dependent degradation of PHB2 [38]. However, the Kla modification of specific proteins also bring positive responses. In some cases, it is discovered that delactylation of some enzymes can also aggravate disease progression. For instance, during the activation of inflammatory macrophages, L-ATP citrate lyase (Acly-L) undergoes Kla modification, which inhibits its enzymatic activity, thereby restricting the productions of IL-1β and IL-6 and attenuating inflammation. In contrast, Acly-S, another isoform of ATP citrate lyase, can not be Kla modified, which leads the expression of excessive IL-1β and IL-6, contributing to rheumatoid arthritis [68]. Another study found that lactylation of fatty acid synthase (FAS) at K673 inhibits its enzymatic activity, reducing fatty acid synthesis, which impairs mitochondrial function and suppresses MPC1 expression through metabolic stress signals (e.g., ROS or AMP/ATP ratio), ultimately alleviating non-alcoholic fatty liver disease symptoms [41]. Kla modifications not only regulate metabolic enzymes but also modulate the activity of E3 ubiquitin ligases. Acetaminophen can induce Kla modification of NEDD4, an E3 ubiquitin ligase, in liver cells, inhibiting its ubiquitination of caspase-11, thereby activating pyroptosis to promote liver injury [42].

Signal transduction-related proteins, mitochondrial-resident proteins, and secretory proteins can also be Kla modified to mediate organ damage. In sepsis, the Kla modification of the signal transduction protein Ezrin induces the activation of nuclear factor κ-B **(**NF-κB) pathway, thus promoting inflammation to induce cell apoptosis and kidney damage [20]. Besides, the Kla modification of mitochondrial fission 1 protein (Fis1) promotes excessive mitochondrial fission, depleting ATP and generating excessive reactive oxygen species (ROS) to induce apoptosis and kidney damage [69]. Furthermore, in liver injury models, the Kla modification of high mobility group box-1 protein (HMGB1) promotes its secretion, leading to macrophage chemotaxis and causing liver damage [39].

In addition to the common L-lactylation, a fraction of proteins can also undergo D-lactylation modification. Under normal metabolic conditions, RelA can be D-lactylated, which suppresses its transcriptional activity, leading to reduction of pro-inflammatory cytokines, preventing excessive immune cell activation and pathological damage caused by inflammation, and restoring immune homeostasis [70]. Therefore, Kla modification may not always promote inflammation but could also have an inhibitory effect.

The aforementioned studies demonstrate that the Kla modifications of functional proteins play crucial roles in the occurrence and development of inflammation. It can exacerbate inflammation and cause organ damage by activating the transcription of pro-inflammatory cytokines and promoting the generation of pro-apoptotic factors, or suppress inflammation by inhibiting the activity of inflammation-related TFs, thus maintaining immune homeostasis.

## The Kla modifications of functional proteins in infectious diseases

**Fig. 2:**
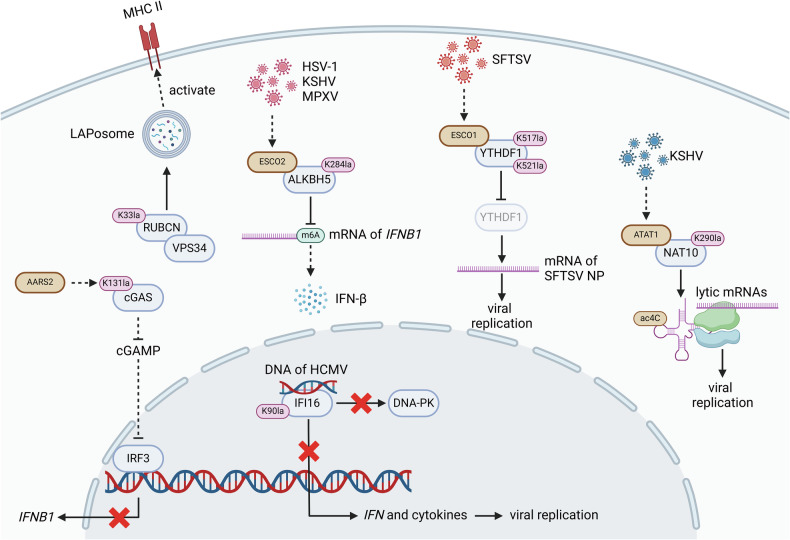
The Kla modifications of functional proteins in infectious diseases. Elevated lactate levels are observed in the microenvironment of some infectious diseases. In tissues with high lactate content, cGAS can be Kla-modified, leading its dysfunction to recognize dsDNA and the production of anti-infective cytokines. Besides, the Kla modification of RUBCN can promote the formation of LAPosome, thereby upregulating MHC Ⅱ to enhance immunological recognition. The Kla modification of IFI16 inhibits its interaction with DNA-PK and prevents the biogenesis of anti-infective cytokines to promote viral replication. KSHV can also induces the Kla modification of NAT10 to enhance stability of the RNA of KSHV, thereby facilitating viral replication. Furthermore, YTHDF1 and ALKBH5, the m6A writer and eraser, can also undergo Kla modifications, resulting in the mRNAs of *IFNB1* and SFTSV NP stabilized to induce distinct effects.

When host cells are infected by pathogens, pattern recognition receptors (PRRs) will be activated, leading to the production of pro-inflammatory cytokines (e.g., Il-1β, TNF-α and IL-6) through the activation of the NF-κB and interferon regulatory factor 3 **(**IRF3) signaling pathways and enhancing the Warburg effect in macrophages and T cells [71–74]. Additionally, pathogens-originated toxins, such as Staphylococcus Aureus α-toxin, can facilitate the formation of plasma membrane pores, leading intracellular ATP depletion and activating the AMPK pathway, thereby initiating glycolysis [75, 76]. Both the pathways above contribute to intracellular lactate production and promote protein Kla modification. Kla modifications of pathogen proteins and host intracellular functional proteins can both promote pathogen replication, thereby facilitating infection and activating inflammation-related signaling pathways to counteract infection. The Kla modifications of pathogen and host intracellular functional proteins play a dual role in infectious diseases (Fig. 2). The roles of the proteins will be introduced in detail in the following sections.

Human cytomegalovirus (HCMV) infection exploits lactate to induce comprehensive Kla modification of host proteins, thereby promoting viral replication. The Kla modification of the viral DNA sensor interferon-γ-inducible protein 16 (IFI16) inhibits the recruitment of DNA-dependent protein kinase (DNA-PK), preventing IFI16-driven virus gene repression and cytokine induction, ultimately promoting viral replication [3]. Similarly, the RNA of Kaposi’s sarcoma-associated herpesvirus (KSHV) induces the Kla modification of N-Acetyltransferase 10 (NAT10) in host cells, enhancing the stability of the RNA of KSHV and increasing its translation efficiency, thereby facilitating viral replication [77]. Besides, viral infection can also induce the Kla modification of the N6-methyladenosine (m6A) reader YTHDF1, reducing its stability and promoting its degradation, thereby increasing viral mRNA stability and facilitating viral replication [44]. Furthermore, viral infection can promote the Kla modification of the m6A demethylase AlkB Homolog 5 (ALKBH5) and enhance its binding to the mRNA of *IFNB* in host cells. However, this effect leads to IFN-β upregulated, thereby inhibiting rather than promoting viral replication [43]. Thus, Kla modification of proteins in host cells might also activate anti-viral effect.

Beyond regulating the activity of RNA-associated proteins to either promote or inhibit viral replication, Kla modification can also modulate the activity of PRRs, reducing immune recognition and thereby facilitating infection. A recent study has shown that a high-lactate environment induced by infection promotes the Kla modification of cyclic GMP-AMP synthase (cGAS), abolishing its DNA-sensing capability. It prevents the activation of the cGAS-STING pathway, reducing immune recognition and impairing the capacity of pathogen clearance [78]. On the contrary, pathogen infected cells would enhance their anti-infective capabilities and promote cell survival in a high-lactate microenvironment through Kla modification of specific functional proteins. For instance, the Kla modification of rubicon autophagy regulator (RUBCN) enhances its interaction with vacuolar protein sorting 34 (VPS34), thereby promoting LAPosome formation and boosting the antibacterial capacity of macrophages [40].

In summary, the levels of lactate increase in pathogen-infected tissues, and pathogens leverage Kla modifications of host RNA-associated enzymes to stabilize their RNA and promote replication. One the one hand, the high-lactate microenvironment also enhances Kla modifications of PRRs, inhibiting their capacities of immune recognition and facilitating immune evasion of pathogens. On the other hand, the host employs Kla modifications of specific proteins in response to the high-lactate microenvironment to strengthen the capacity of pathogen clearance, thus protecting the host from infection. Therefore, Kla modification plays a crucial role in the dynamic interplay between host and pathogen.

## The Kla modifications of functional proteins in neurological diseases

**Fig. 3:**
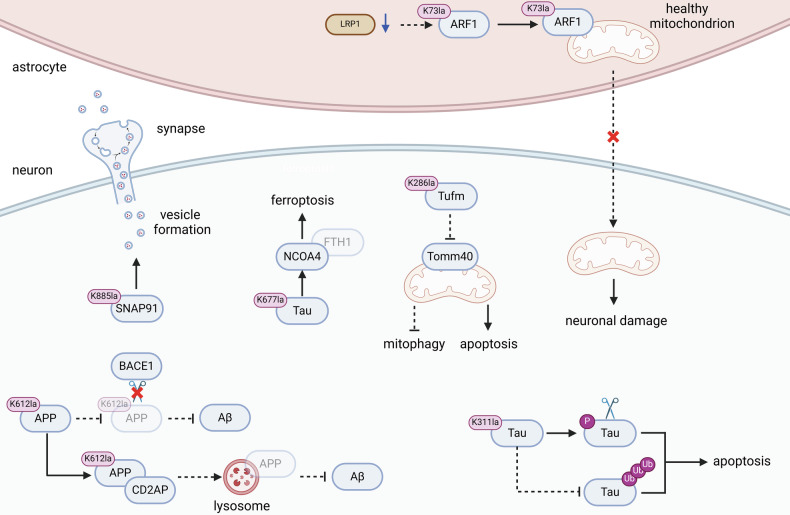
The Kla modifications of functional proteins in neurological diseases. The Kla modifications of functional proteins play both accelerative and suppressive roles in neurological diseases. The Kla-modified Tau can induce apoptosis and ferroptosis of neuron, thereby promoting the progression of AD. The Kla modification of Tufm can attenuate its interaction with Tomm40, inhibiting mitophagy to induce neuronal apoptosis. The vesicle-resident protein ARF can also be Kla-modified to inhibit the transmitting of healthy mitochondria from astrocytes to neurons, resulting in neuronal damage. On the contrary, the Kla modification of APP can inhibit the accumulation of Aβ, relieving the progression of AD. The Kla-modified SNAP91 can also promote vesicle formation and remodeling the shape of synapse to relieve anxiety disorder.

Neurological diseases are primarily categorized into four major types: neurodegenerative diseases, cerebrovascular diseases, neuroimmune diseases, and epilepsy [79–82]. Age-related risk factors significantly amplify the incidence of neurodegenerative diseases and cerebrovascular disorders in elderly populations, with these conditions collectively driving progressive disability and high mortality rates globally [83, 84]. According to the Global Burden of Disease data, the combined annual mortality from cerebrovascular disorders (6.5 million) and major neurodegenerative diseases (2.7 million) exceeds 9 million deaths globally, representing the third leading cause of death after cardiovascular diseases and cancers [83, 84]. Globally, approximately 6.6 million deaths annually are attributable to stroke, with acute mortality rates ranging from 10% to 20% for all stroke types and escalating to 30%-40% for hemorrhagic subtypes. Among survivors, over 50% experience disabling complications, including hemiplegia and aphasia [83, 85–87]. Besides, neurodegenerative diseases, including Alzheimer’s disease (AD), Parkinson’s disease (PD), and amyotrophic lateral sclerosis (ALS), though associated with lower acute mortality rates, pose more profound long-term harm [88–90].Particularly, ALS, being the most severe of the five major neurodegenerative diseases, still lacks an effective treatment, with a short survival period and a mortality rate close to 100% [91]. The evidence above highlights the significant threat of neurological diseases to human health, underscoring the urgency of research into their pathogenesis and treatments. Therefore, studies on the roles of Kla modifications of functional proteins in neurological diseases are being actively pursued, with Fig. 3 illustrating the impact of the Kla modifications of key protein in the diseases above.

Recent studies have shown that the Kla modifications of functional proteins are closely related to the progression of AD. In the pathological tissues of AD, accumulated β-amyloid plaques (Aβ) excessively phosphorylate Tau, leading to the formation of neurofibrillary tangles (NFTs), which results in neuronal dysfunction or death [92–94]. A recent study demonstrated elevated lactate levels in the pathological tissues of AD compared to healthy controls, driving Kla modification of Tau at K331. This lactylation enhances Tau phosphorylation and cleavage by altering Tau’s conformation, increasing its susceptibility to GSK-3β and caspase-2. Additionally, lactylation at K331 competes with ubiquitination at the same lysine residue, reducing ubiquitin-dependent degradation of Tau via the ubiquitin-proteasome system, thereby promoting pathological Tau accumulation and accelerating AD progression [95]. Furthermore, Tau can also be lactylated at K677, which regulates iron metabolism-related factors such as NCOA4 and FTH1, promoting ferroptosis, exacerbating neuronal damage, impairing learning and memory abilities, thereby advancing the progression of AD [96]. The amyloid precursor protein (APP) in neurons of AD patients can also be lactylated. However, the Kla modification level of APP in neurons of patients is low, leading to disfunction of its degradation via the endosome-lysosome pathway. When the level of lactate further increases, the Kla modification of APP is enhanced, promoting its internalization from the plasma membrane to the endosome, followed by CD2AP-mediated endosome-lysosome degradation, which reduces Aβ production and accumulation, thereby improving neurocognition [97]. This study demonstrates that Kla modifications of functional proteins in neurons can also alleviate symptom of AD. However, the Kla modification of Tau plays a more significant role in facilitating AD progression.

The Kla modifications of functional proteins are also closely associated with stroke. ARF1, the protein involves in vesicular transport of astrocytes, can also undergo Kla modification while low-density lipoprotein receptor-related protein 1 (LRP1) is inhibited, which inhibits its function of transmitting healthy mitochondria from astrocytes to neurons, thereby leading to mitochondrial dysfunction in neurons and inducing stroke [98]. This study demonstrates that Kla modifications of functional proteins can regulate the intercellular transmission of organelles, thereby modulating the physiological and biochemical processes of neighboring cells.

The Kla modifications of functional proteins also play regulatory roles in other neurological diseases. Similar to the aforementioned diseases, total Kla modification levels of functional proteins are also elevated in pathological tissues of traumatic brain injury (TBI). Tufm, a key factor regulating mitophagy, has been found to be lactylated in damaged cells of TBI. The Kla modification of Tufm inhibits its interaction with Tomm40 on mitochondria and suppresses Tufm-mediated mitophagy, further promoting mitochondrial-induced neuronal apoptosis [99]. Additionally, lactate produced by anaerobic exercise can enhance the Kla modification of synaptosome-associated protein 91 (SNAP91) in neurons, modulating synaptic structure in the medial prefrontal cortex (mPFC) and promoting neuronal motility, thus alleviating anxiety disorder [100].

The abovementioned studies confirm that the Kla modifications of functional proteins play vital roles in astrocytes and neurons to induce neurological diseases.

## The Kla modifications of functional proteins in cardiovascular diseases

Cardiovascular diseases (CVD) primarily include coronary atherosclerotic heart disease (CHD), hypertension-related hypertensive heart disease, heart failure (HF), arrhythmias, valvular heart disease, peripheral artery disease and aortic-related disorders [101–104]. CVD is the leading cause of death among the elderly and the most common cause of death globally, with an annual incidence of approximately 20 million cases, similar to that of malignant tumors [105]. However, the annual deaths of CVD reach 18.6 million, accounting for 32% of global deaths, which is significantly higher than the 10 million deaths caused by malignant tumors [105–107]. CVD progresses quickly with an extremely high mortality rate during the acute phase [108, 109]. Acute coronary syndrome (ACS), acute aortic dissection (AAD), malignant arrhythmias, and acute decompensated heart failure (ADHF) exhibit the highest mortality rates within 30 days from onset of diseases, ranging from 10% to 50% [110–113]. Even with treatment and successful recovery from the acute phase, patients may suffer from complications such as ventricular aneurysms, ventricular arrhythmias, renal insufficiency, and spinal ischemia, which significantly impact their quality of life [114]. Although the management of CVD is indispensable for lifesaving interventions and prognosis optimization, preventive strategies retain paramount importance in interrupting the pathogenic cascade at its upstream origins to fundamentally block disease progression. Therefore, studies on the pathogenesis of CVD provide a theoretical foundation for better prevention and treatment strategies.

**Fig. 4:**
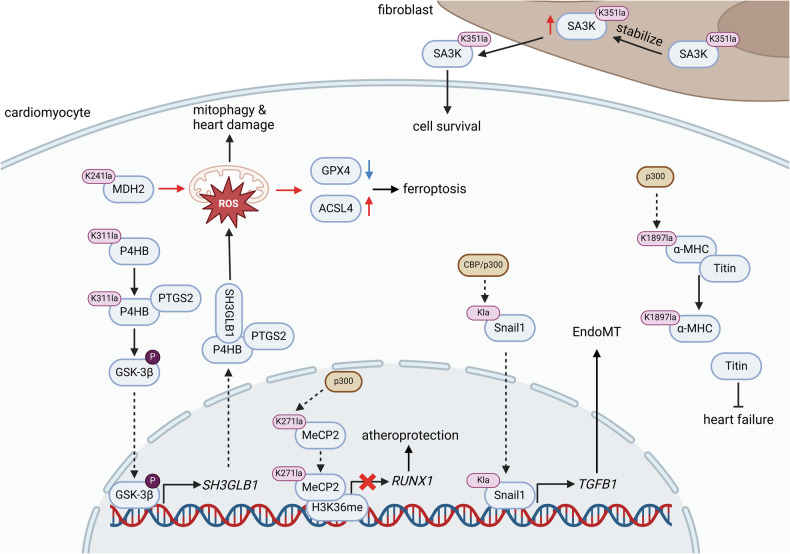
The Kla modifications of functional proteins in cardiovascular diseases. The Kla modifications of functional proteins also play significant roles in the progression of cardiovascular diseases. The Kla modification of MDH2 and P4HB in cardiomyocyte can promote the accumulation of mitoROS to induce its ferroptosis and mitophagy. The Kla-modified α-MHC can also induce heart failure via regulating cell skeleton depolymerization. The Kla modifications of MeCP2 and Snail1 can also exerts protective and harmful effects to cardiomyocyte through modulating transcription of specific genes. Furthermore, the Kla modification of SA3K in fibroblasts could facilitate the survival of neighboring cardiomyocytes.

As illustrated in Fig. 4, Kla modifications of functional proteins in high-lactate microenvironments play both promotive and inhibitory roles in CVD pathogenesis. Specifically, cellular stresses, such as hypoxia, inflammation, and metabolic disorders, contribute to the development of CVD, leading to myocardial ischemia, HF, and atherosclerosis [115–119]. Due to the upregulation of hypoxia-inducible factor 1-alpha (HIF-1α) and the activation of glycolytic pathways, the lactate level in microenvironment of CVD tissues is higher than that in normal conditions, resulting in Kla modifications of functional proteins, which induce both promotive and inhibitory effects on the onset and progression of CVD [120–122]. A recent study has indicated that Snail1 can be lactylated, which promotes its nuclear translocation, activating the TGF-β/Smad2 pathway, and induces endothelial-mesenchymal transition (EndoMT), leading to reduced coronary artery elasticity and impaired angiogenesis, thereby contributing to myocardial infarction [123]. In cardiomyocytes, prolyl 4-hydroxylase-β subunit (P4HB), a protein disulfide isomerase, undergoes Kla modification at K311 during DNA damage, enhancing its interaction with cyclooxygenase 2 (PTGS2), the key factor involves in synthesis of prostaglandin and regulation of oxidative stress. This interaction promotes mitochondrial reactive oxygen species (mitoROS) generation by activating the PTGS2-SH3GLB1 axis, which disrupts mitochondrial complex function and triggers NDP52-mediated mitophagy, exacerbating radiation-induced heart disease (RIHD) [124]. The effect above induces mitophagy, contributing to myocardial injury. Additionally, ischemia-reperfusion injury (IRI) after surgery can upregulate pyruvate dehydrogenase kinase 4 (PDK4), activating glycolysis and promoting the Kla modification of malate dehydrogenase 2 (MDH2), which damages myocardial mitochondrial function and induces ferroptosis of cardiomyocytes [125]. This effect can be mitigated by dexmedetomidine treatment, improving mitochondrial function and protecting cardiomyocytes.

In high-lactate microenvironments, Kla modifications of functional proteins also play protective roles in cardiomyocytes. As previously mentioned, IRI causes an increase of intracellular lactate, leading to the Kla modification of serine protease inhibitor Serpina3k (SA3K), which enhances its stability to increase the protein level. The upregulated SA3K primarily protects cells from ischemia-reperfusion-induced apoptosis through paracrine signaling and by activating cardioprotective reperfusion injury salvage kinase and survivor activating factor enhancement pathways [126]. Moreover, Kla modification of α-myosin heavy chain (α-MHC) promotes its interaction with the cardiac myocyte structural protein titin, thereby maintaining the structure of cardiomyocytes. In patients with HF, the lactylation level of α-MHC is reduced, leading to compromised cardiac structure and function [127]. Furthermore, Kla modification also plays a crucial role in the prevention of cardiovascular diseases. Lactate generated during anaerobic exercise can induce the Kla modification of methyl-CpG binding protein 2 (MeCP2) to promote its interaction with histone H3 lysine 36 trimethylation (H3K36me3), resulting in chromatin accessibility increased and the transcriptional activity of the RUNX1 inhibited. The effect above facilitates the polarization of pro-repair M2 macrophages to prevent atherosclerosis [128].

Similar to the role of Kla modification in neurological diseases, Kla modifications of functional proteins exhibit Janus-faced roles in cardiovascular diseases. These findings provide important theoretical evidence for the prevention and treatment of cardiovascular diseases.

## The Kla modifications of functional proteins in cancer

Since the mid-20^th^ century, cancer has progressively emerged as the second leading cause of death worldwide, posing a persistent threat to global public health [129, 130]. Although the annual global incidence of cancer is comparable to that of cardiovascular diseases, with about 10 million deaths annually, lower than that of cardiovascular diseases, the incidence of cancer has been increasing annually due to longer life expectancies and detrimental lifestyle patterns [107, 131, 132]. Statistics reveal that the global annual incidence of new cancer cases tripled from 6.4 million in 1980 to 19.3 million in 2020, demonstrating a sharp increase in yearly diagnosis rates over the four-decade period [107, 133]. In contrast to cardiovascular diseases, which predominantly affect older populations, cancer is increasingly characterized by a younger-onset trend [107, 134, 135]. Emerging evidence suggests that this shift in epidemiological patterns may be linked to lifestyle-related risk factors prevalent among younger generations, including circadian rhythm dysregulation, prolonged exposure to high-sugar, high-fat and aflatoxin contaminated diets, and sustained psychosocial stressors such as occupational anxiety and social media-induced mental load [136–140]. Therefore, studies on cancer prevention and treatment have profound significance for preserving health security.

**Fig. 5:**
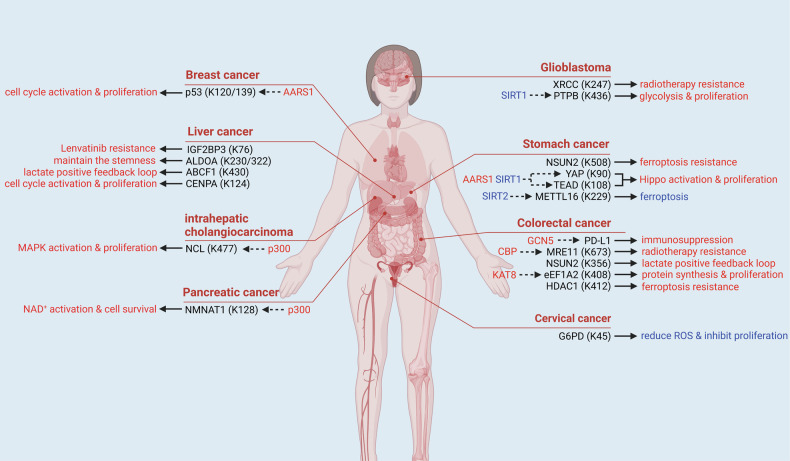
The Kla modifications of functional proteins in cancer. Oncoproteins and tumor suppressors can also undergo Kla modifications. The Kla-modified sites of proteins and their corresponding lactytransferases (red) and delactylases (blue) are presented. The Kla modifications of indicated proteins can induce radiotherapy and immunotherapy resistance, cell death resistance, production of lactate and activation of cell growth-related signaling pathways (red). Meanwhile, the Kla modifications of METTL16 and G6PD can also attenuate cancer progression via reducing ROS production and inhibiting ferroptosis (blue).

Figure 5 illustrates the multifaceted roles of lactate and Kla modifications in cancer progression, including metabolic reprogramming and immune evasion. The heightened proliferative activity of cancer cells drives an elevated demand for glucose, leading to significant upregulation of glucose transporter 1 (GLUT1) on the cell surface to enhance glucose uptake [141–143]. Concurrently, the Warburg effect, a metabolic hallmark of malignancies, induces marked activation of the glycolytic pathway in cancer cells, resulting in substantial lactate production despite adequate oxygen availability [35, 144, 145]. Lactate generated by cancer cells is partially exported into the extracellular spaces, where it suppresses CD8^+^ T cell proliferation, cytotoxic activity, and IFN-γ secretion, thereby impairing immune surveillance and fostering tumor immune evasion [146].

Hypoxia, a hallmark of cancer, significantly enhances lactate production via the Warburg effect, thereby driving Kla modifications. Under hypoxic conditions, cancer cells upregulate HIF-1α, activating glycolytic enzymes such as LDHA, leading to elevated lactate levels in the tumor microenvironment [144]. This lactate accumulation induces Kla modifications of functional proteins, affecting transcription factors, DNA repair proteins, and immune checkpoint proteins, thereby promoting tumor proliferation, metastasis, and immune evasion [144]. Hypoxia-induced lactylation plays a pivotal role in cancer progression by regulating key signaling pathways and metabolic reprogramming, with detailed mechanisms discussed later in this section [45, 147, 148].

Intracellularly, lactate accumulation activates HIF-1α-mediated transactivation of programmed death-ligand 1 (PD-L1), establishing additional immune tolerance mechanisms [149, 150]. Simultaneously, lactate functions as a signaling metabolite to stimulate NF-κB pathway activation, which drives epithelial-mesenchymal transition (EMT) and enhances vascular endothelial growth factor (VEGF) secretion [151, 152]. The processes above synergistically promote angiogenic remodeling of the tumor microenvironment (TME). Consequently, lactate serves as a critical regulator in both tumorigenesis and immune evasion. Given its pivotal role within the TME, Kla modifications of functional proteins in malignancies have been most extensively characterized. These Kla-mediated PTMs exert profound regulatory effects on multiple oncogenic processes, including tumor proliferation and metastasis, metabolic reprogramming, DNA damage repair, as well as resistance mechanisms to immunotherapy and targeted therapy [45, 147, 153–157].

The Kla modifications of oncogenic TFs are pivotal in driving tumor growth. A recent study revealed that in gastric cancer (GC) cells, TEA domain family member (TEAD) and its coactivator Yes-associated protein (YAP) undergo lactylation, enhancing the stability and transcriptional activity of the YAP-TEAD complex, thus activating the expression of aminoacyl-tRNA synthetase 1 (AARS1), which, beyond its canonical role in protein synthesis, functions as a lactyltransferase. AARS1 senses elevated lactate levels in the tumor microenvironment and translocates to the nucleus, where it further lactylates YAP and TEAD, reinforcing their interaction and promoting tumorigenesis in GC [148]. The E2 ubiquitin ligase ABC transporter family C member 1 (ABCF1) can also be Kla modified in GC, translocating into the nucleus to act as a TF, binding to the promoter of *KDM3A*, and promoting its transcription. The transactivation of *KDM3A* activates the KDM3A-H3K9me2-HIF-1α axis to enhance HCC growth [153]. Additionally, the Kla modifications of some transcriptional co-factors can activate TFs through enhancing their interactions. In HCC cells, the Kla modification of centromere protein A (CENPA) facilitates the formation of CENPA-YY1 complex, which activates the transcription of *CCND2* and *NRP2* genes, promoting tumor growth, metastasis, and angiogenesis in the TME [158]. The Kla modifications also promote the nuclear translocation of nuclear proteins without transcriptional activities. In pancreatic adenocarcinoma (PAAD), nicotinamide mononucleotide adenylyltransferase 1 (NMNAT1) is modified by Kla, enhancing its nuclear localization and enzymatic activity, thereby supporting the nuclear NAD^+^ salvage pathway and facilitating cancer progression [159]. Besides, the Kla modifications of DNA damage repair-associated proteins can enhance their activities to maintain genomic stability in cancer cells and promote tumorigenesis. In glioblastoma stem cells (GSCs), the Kla modification of X-ray repair cross-complementing protein 1 (XRCC1) promotes its binding with importin-α to facilitate its nuclear translocation, thus improving DNA damage repair capacity and radiotherapy resistance [147]. Furthermore, radiotherapy can promote the Kla modification of meiotic recombination 11 homolog (MRE11) by activating ATM phosphorylation, enhancing the DNA-binding ability of MRE11, mediating DNA damage repair to induce radiotherapy resistance [160].

Some recent studies have shown that Kla modifications in tumor cells can impact RNA modifications, thereby regulating the stability and translation of specific RNAs. This pathway also plays an essential role in tumorigenesis. In the TME of HCC patients, the Kla modification of insulin-like growth factor binding protein 3 (IGFBP3), the m6A reader YTH domain family protein 2 (YTHDF2) binding protein, can enhance its interaction with the mRNAs of *PCK2* and *NRF2*, thereby transactivating the genes to promote lenvatinib resistance [156]. In colorectal cancer (CRC), the Kla modification of histone deacetylase 1 (HDAC1) reduces the levels of RNA demethylases fat mass and obesity-associated protein (FTO) and ALKBH5, increasing m6A modification on the mRNA of *FSP1* to upregulate ferroptosis inhibitor protein ferroptosis suppressor 1 (FSP1), inhibiting ferroptosis and promoting tumor growth [161]. However, the Kla modifications of m6A-related enzymes may also inhibit tumor growth. The atypical m6A reader methyltransferase-like 16 (METTL16) can be lactylated in GC, promoting the m6A modification of the mRNA of *FDX1*, thus facilitating ferroptosis [162].

In addition to the previously mentioned m6A modification, mRNA can also undergo 5-methylcytosine (m5C) modification, which, analogous to m6A, has been demonstrated to regulate mRNA stability [163–165]. Moreover, m5C modifications also influence mRNA splicing and subcellular localization. In cholangiocarcinoma (CG), the m5C methyltransferase can be lactylated at K508 to significantly enhance its activity, which enhances the stability of the mRNA of *GCLC*, leading to the upregulation of GCLC, which induces the production of glutathione (GSH). The effect above can effectively suppress cellular ferroptosis by scavenging free radicals and reducing lipid peroxidation levels [166]. Furthermore, in CRC, NSUN2 could also be lactylated at K356, significantly enhancing its interaction with the mRNA of *ENO1*, and the upregulation of enolase 1, the protein encoded by *ENO1*, promotes glycolysis to generate more lactate and facilitate CRC progression [154].

Additionally, the Kla modifications of functional proteins also exert regulatory roles in RNA splicing and processing. High lactate microenvironment causes the Kla modification of polypyrimidine tract-binding protein 1 (PTBP1), the RNA processing protein, in GSCs, which inhibits its degradation by reducing its interaction with E3 ubiquitin ligase TRIM21. It elevates the expression of the mRNA of *PFKFB4*, the gene encoding key glycolytic enzyme, which further generates lactate, thus promoting glioma progression [155]. Another study suggests that in intrahepatic cholangiocarcinoma (ICC), nucleolin (NCL) undergoes Kla modification, which inhibits the alternative splicing of the precursor mRNA of *MADD*, thereby increasing its integrity and stability, upregulating mitogen-activated protein kinase-activated protein kinase (MADD) and activating the mitogen-activated protein kinase (MAPK) signaling pathway, facilitating ICC growth [167].

The Kla modifications of functional proteins can also directly regulate protein synthesis in cancer cells. In CRC, translation elongation factor eEF1A2 is lactylated to promote its mediation of ribosomal translation, which enhances total protein synthesis, leading to cell division and tumor proliferation [168].

Kla modifications also play critical roles in tumor immune evasion. In CRC, the lactylation of PD-L1 inhibits degradation via ubiquitin-proteasome pathway, and stabilizes cell surface PD-L1. Upregulated PD-L1 suppresses the activity of CD8^+^ T cells and attenuates their ability in impairing the immune surveillance and anti-tumor capabilities [45].

In addition to their previously described functions, Kla modifications of functional proteins play critical roles in maintaining cancer stemness, inducing cellular oxidative stress, and promoting cell cycle progression in tumors [169–171]. These studies demonstrate that while histone Kla modifications exert broad regulatory effects in malignancies, the Kla modifications of functional proteins are equally essential for tumorigenesis. The pleiotropic effects of histone Kla modifications, mediated through extensive gene network regulation, render them suboptimal therapeutic targets [33, 172]. In contrast, the Kla modifications of functional proteins exhibit superior target specificity due to their focused action on defined functional pathways, positioning them as more viable candidates for precision oncology. Future development of pharmacological agents targeting the Kla modifications of functional proteins holds promising therapeutic potential for clinical cancer management.

## Conclusions and future perspectives

Kla modification, a recently discovered PTM, is currently under intensive investigations for its functions and mechanisms [173, 174]. Numerous studies have demonstrated that Kla modification plays a crucial role in the pathogenesis and development of various diseases [175–177]. Cellular stresses induced by inflammation, microbial infections, cellular damage, neurodegenerative diseases, cardiovascular diseases and the Warburg effect would facilitate lactate production and its accumulation in pathological tissues, resulting in significant upregulation of total Kla modifications of proteins involved in these diseases [78, 97, 98, 127, 145, 178]. Some early studies have shown that the Kla modifications of histone H3 and H4 subunits are the most prominent in total cellular proteins; however, due to their comprehensive regulatory effects on gene transcription, developing inhibitors targeting these modification sites could suppress broad transcriptional activities, making them unsuitable as drug targets [15, 17, 31, 179].

In a high-lactate microenvironment, the Kla modifications of functional proteins predominantly promote disease pathogenesis and progression through multiple mechanisms, including transactivation of pro-inflammatory cytokines, modification of specific mRNAs to enhance their stability and facilitate translation, induction of mitochondrial damage, enhancement of DNA damage repair in cancer cells, and participation in intracellular signal transduction pathways [38, 68, 147, 155, 167]. Generally, Kla modifications occur predominantly in pathological tissues with high lactate level, accelerating the disease progression [180–182]. However, as the organism possesses intrinsic mechanisms to counteract disease progression, Kla modifications may also inhibit or impede pathological development under certain conditions [98, 125]. Consequently, Kla modifications exhibit a dual regulatory role in disease contexts, either promoting or suppressing disease progression depending on specific cellular environments or pathological stages.

**Fig. 6:**
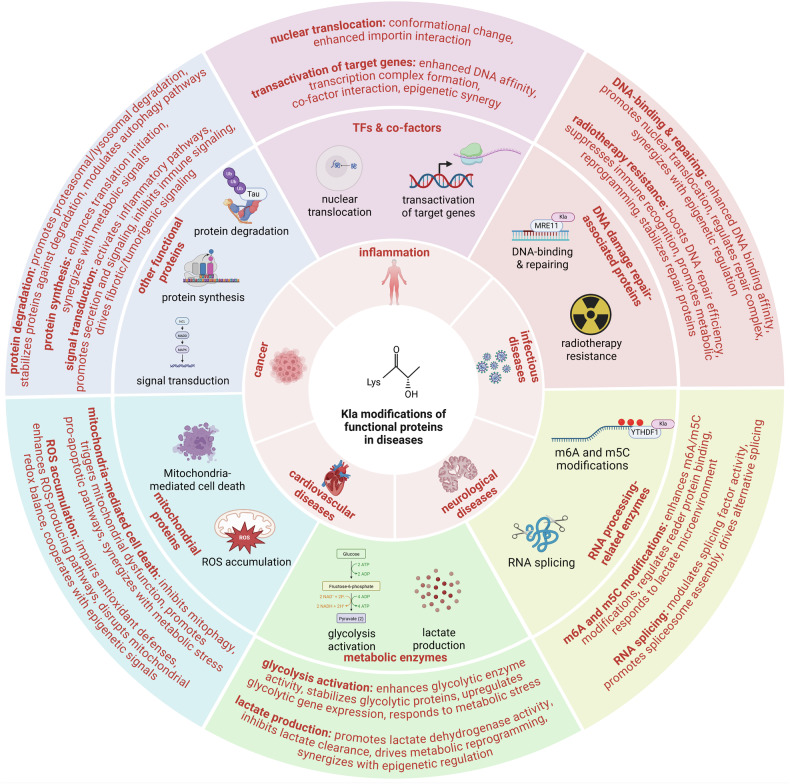
The hallmarks of Kla-modified functional proteins in diseases. Functional proteins undergo Kla modifications during the processes of various diseases, including inflammation, infectious diseases, neurological diseases, cardiovascular diseases and cancer. The Kla modifications can facilitate the nuclear translocation of TFs, and enhance their interactions with promoters to transactivate specific genes. The Kla modifications can also promote DNA damage repairing to induce radiotherapy resistance of cancer cells. Besides, the Kla modifications of RNA methylation-related enzymes can also stabilize mRNAs, facilitate their translations, or promote RNA splicing, thus accelerating protein synthesis. The Kla-modified metabolic enzymes can constantly activate glycolysis to promote lactate production and form the lactate positive feedback loop. Moreover, the Kla modifications of some mitochondrial proteins can cause the accumulation of mitoROS, and trigger mitochondria-mediated cell death. In addition to the effects above, the Kla modifications of specific proteins can also regulate their synthesis, degradation and activities to modulate signal transduction.

Mass spectrometry analysis of global protein Kla modifications and immunoblotting analysis have revealed that, in addition to well-documented histone Kla modifications, functional proteins exhibit substantial Kla modification abundance [78, 183]. Subsequent studies have corroborated that these Kla modifications of functional proteins play significant roles in disease progression (Fig. 6), which can be categorized as follows:

1. **TFs and their co-factors:** We have collected and analyzed the studies of lactylation and discovered that this class of proteins represents the most prevalent targets of Kla modifications, second only to histones. During disease progression, Kla modifications on specific TFs and co-regulators facilitate the expression of pro-pathogenic genes, thereby driving disease pathogenesis and advancement.
2. **DNA damage repair-associated proteins:** In diseases such as cancer, the genome experiences DNA replication stress, leading to damage or even strand breaks that ultimately promote cell death [184–186]. It necessitates the recruitment of DNA damage repair proteins to restore genomic integrity [187–189]. Notably, Kla modifications on DNA damage repair-associated proteins facilitate their nuclear translocation and enhance DNA-binding affinity, thereby promoting efficient damage repair and ensuring cellular survival [147, 160]. Paradoxically, this pro-survival mechanism also confers tumor resistance to radiotherapy in clinical contexts.
3. **RNA processing-related proteins:** The m6A and m5C RNA modifications are identified on the c-terminal of coding DNA sequences (CDS) and 3’ untranslated regions (UTRs) of mRNAs, which could enhance the stabilities of specific proteins and facilitate their translation [190–192]. Several studies have confirmed that enzymes involved in m6A and m5C RNA modifications can be significantly Kla-modified, leading to enhanced stability or increased translation efficiency of specific mRNAs [156, 166]. Besides, the Kla modification of RNA-splicing-associated enzyme can also facilitate the expression of oncoproteins, resulting in the activation of MAPK signaling pathway to promote cancer progression [167]. The effects above upregulate specific proteins via enhancing the stability of mRNAs or promoting their translation, thus accelerating disease progression.
4. **Metabolic enzymes:** Intracellular lactate accumulation primarily results from activated glycolytic pathways [193–195]. The resultant high-lactate microenvironment further induces Kla modifications on glycolysis-related enzymes [41, 169]. The Kla modifications of these enzymes enhance glycolytic activity, leading to increased lactate production and establishing a self-reinforcing positive feedback loop. This cycle amplifies both lactate levels and Kla modifications on other functional proteins, thereby accelerating disease progression.
5. **Mitochondrial proteins:** Mitochondria not only produce energy through oxidative phosphorylation but are also central to the intrinsic pathway of apoptosis [196–199]. The Kla modifications of mitochondrial proteins enhance their oxidative phosphorylation activity, leading to excessive ROS production, while simultaneously promoting mitochondrial-mediated apoptotic pathways [99, 124]. Both effects synergistically induce tissue damage.
6. **Other functional proteins:** The Kla modifications also target ubiquitin ligases, protein synthesis machinery components, and signaling transducers, thereby exerting regulatory control over disease pathogenesis and progression [20, 42, 168]. It is deemed that a considerable number of functional proteins may harbor Kla modifications, yet this field remains largely uncharted, presenting a vast research frontier that urgently demands further systematic exploration.

For scientists interested in exploring the Kla modifications of proteins, several studies provide comprehensive methodologies and reagents for detecting and characterizing them. LC-MS/MS has been instrumental in identifying Kla sites, as demonstrated in the initial discovery of histone lactylation [7]. Additionally, anti-L-Kla antibodies are widely used for immunoblotting, immunoprecipitation, and immunofluorescence to detect Kla modifications across histone and non-histone proteins. Anti-L-Lactyl Lysine Rabbit mAb (PTM-1401RM, PTM Bio, Chicago, IL, USA) is currently a highly cited and high-quality antibody used for lactylation detection [98, 160, 200]. High-throughput lactylome profiling and CRISPR-based gene editing further enable the study of Kla dynamics and functional roles of lactyltransferases [78]. These methodologies, including quantitative proteomics, site-specific mutagenesis, and chromatin immunoprecipitation, offer robust tools for investigating molecular mechanisms and therapeutic potential of Kla modifications in various diseases.

Although studies on the Kla modifications of functional proteins have been rapidly advancing, the aspects below remain poorly understood. Firstly, while the Kla modifications of many functional proteins have been identified through LC-MS/MS, the biological effects of these modifications remain unclear. Secondly, the writers and erasers governing lactylation and delactylation of regulatory proteins remain incompletely characterized. Identifying these regulatory enzymes and elucidating their substrate specificity will facilitate the development of therapeutic targets for diseases above. Thirdly, proteins susceptible to Kla modification under pathological conditions with high lactate levels are likely to exhibit functional synergies in cellular stress adaptation mechanisms. Deciphering these cooperative interactions could deepen our understanding of Kla modifications in diseases. Finally, in contrast to histone Kla modifications that drive broad transcriptional reprogramming, Kla modifications of functional proteins often exert more discrete functional effects. Targeting Kla modifications of functional proteins may enable precision therapeutics with enhanced efficacy and reduced adverse effects [201].

Despite the emerging significance of Kla modifications in disease pathogenesis, its clinical translation faces multiple challenges. The dynamic regulation of lactylation, driven by variable lactate levels across tissues and disease states, hinders the development of consistent biomarkers [202]. Additionally, the broad specificity of Kla modifications across diverse proteins complicates pinpointing critical modification sites for precise therapeutic targeting [203]. Current detection methods, such as mass spectrometry, remain technically complex and impractical for routine clinical use, necessitating simpler assays [204]. Furthermore, the incomplete understanding of functional consequences of Kla modifications limits the design of precise interventions [205]. Finally, therapeutic modulation of lactylation lacks specificity and clinical validation, requiring further research to unlock its potential in practice [206].

Although Kla-targeted therapies show promising therapeutic potential, they remain in nascent stages of development. Further mechanistic investigations and translational studies are imperative to advance these strategies toward clinical implementation, ultimately expanding the arsenal for disease intervention.

## References

[1] Solis ER, Jameson JM. Skin deep: Epithelial cell metabolism and chronic skin inflammation. Immunity. 2024;57:1451–3. 38986439 10.1016/j.immuni.2024.06.004

[2] Bei Y, Zhu Y, Zhou J, Ai S, Yao J, Yin M, et al. Inhibition of Hmbox1 Promotes Cardiomyocyte Survival and Glucose Metabolism Through Gck Activation in Ischemia/Reperfusion Injury. Circulation. 2024;150:848–66. 38708602 10.1161/CIRCULATIONAHA.123.067592

[3] Tyl MD, Merengwa VU, Cristea IM. Infection-induced lysine lactylation enables herpesvirus immune evasion. Sci Adv. 2025;11:s6215. 10.1126/sciadv.ads6215 PMC11708889 39772686

[4] Zhang X, Peng L, Kuang S, Wang T, Wu W, Zuo S, et al. Lactate accumulation from HIF-1α-mediated PMN-MDSC glycolysis restricts brain injury after acute hypoxia in neonates. J Neuroinflamm. 2025;22:59. 10.1186/s12974-025-03385-8 PMC11871681 40025545

[5] Zhang X, Wang Y, Li H, Wang DW, Chen C. Insights into the post-translational modifications in heart failure. Ageing Res Rev. 2024;100:102467. 39187021 10.1016/j.arr.2024.102467

[6] Tufail M, Jiang C, Li N. Altered metabolism in cancer: insights into energy pathways and therapeutic targets. Mol Cancer. 2024;23:203. 39294640 10.1186/s12943-024-02119-3 PMC11409553

[7] Zhang D, Tang Z, Huang H, Zhou G, Cui C, Weng Y, et al. Metabolic regulation of gene expression by histone lactylation. Nature. 2019;574:575–80. 31645732 10.1038/s41586-019-1678-1 PMC6818755

[8] Jackson V. Studies on histone organization in the nucleosome using formaldehyde as a reversible cross-linking agent. Cell. 1978;15:945–54. 569554 10.1016/0092-8674(78)90278-7

[9] Richmond TJ, Finch JT, Rushton B, Rhodes D, Klug A. Structure of the nucleosome core particle at 7 Å resolution. Nature. 1984;311:532–7. 6482966 10.1038/311532a0

[10] Methot SP, Padeken J, Brancati G, Zeller P, Delaney CE, Gaidatzis D, et al. H3K9me selectively blocks transcription factor activity and ensures differentiated tissue integrity. Nat Cell Biol. 2021;23:1163–75. 34737442 10.1038/s41556-021-00776-w PMC8572725

[11] Binder M, Carr RM, Lasho TL, Finke CM, Mangaonkar AA, Pin CL, et al. Oncogenic gene expression and epigenetic remodeling of cis-regulatory elements in ASXL1-mutant chronic myelomonocytic leukemia. Nat Commun. 2022;13:1434. 35301312 10.1038/s41467-022-29142-6 PMC8931048

[12] Sheu S, Upadhyayula S, Dupuy V, Pang S, Deng F, Wan J, et al. A serotonergic axon-cilium synapse drives nuclear signaling to alter chromatin accessibility. Cell. 2022;185:3390–407. 36055200 10.1016/j.cell.2022.07.026 PMC9789380

[13] Cao Y, Zhang X, Akerberg BN, Yuan H, Sakamoto T, Xiao F, et al. In Vivo dissection of chamber-selective enhancers reveals estrogen-related receptor as a regulator of ventricular cardiomyocyte identity. Circulation. 2023;147:881–96. 36705030 10.1161/CIRCULATIONAHA.122.061955 PMC10010668

[14] Zhu R, Ye X, Lu X, Xiao L, Yuan M, Zhao H, et al. ACSS2 acts as a lactyl-CoA synthetase and couples KAT2A to function as a lactyltransferase for histone lactylation and tumor immune evasion. Cell Metab. 2025;37:361–76. 39561764 10.1016/j.cmet.2024.10.015

[15] Li X, Chen M, Chen X, He X, Li X, Wei H, et al. TRAP1 drives smooth muscle cell senescence and promotes atherosclerosis via HDAC3-primed histone H4 lysine 12 lactylation. Eur Heart J. 2024;45:4219–35. 39088352 10.1093/eurheartj/ehae379 PMC11481199

[16] Li L, Dong J, Xu C, Wang S. Lactate drives senescence-resistant lineages in hepatocellular carcinoma via histone H2B lactylation of NDRG1. Cancer Lett. 2025;616:217567. 39978571 10.1016/j.canlet.2025.217567

[17] Raychaudhuri D, Singh P, Chakraborty B, Hennessey M, Tannir AJ, Byregowda S, et al. Histone lactylation drives CD8+ T cell metabolism and function. Nat Immunol. 2024;25:2140–51. 39375549 10.1038/s41590-024-01985-9 PMC13211864

[18] Liu R, Ren X, Park YE, Feng H, Sheng X, Song X, et al. Nuclear GTPSCS functions as a lactyl-CoA synthetase to promote histone lactylation and gliomagenesis. Cell Metab. 2025;37:377–94. 39642882 10.1016/j.cmet.2024.11.005 PMC11798710

[19] Li F, Si W, Xia L, Yin D, Wei T, Tao M, et al. Positive feedback regulation between glycolysis and histone lactylation drives oncogenesis in pancreatic ductal adenocarcinoma. Mol Cancer. 2024;23:90. 38711083 10.1186/s12943-024-02008-9 PMC11071201

[20] Qiao J, Tan Y, Liu H, Yang B, Zhang Q, Liu Q, et al. Histone H3K18 and Ezrin Lactylation Promote Renal Dysfunction in Sepsis-Associated Acute Kidney Injury. Adv Sci. 2024;11:2307216. 10.1002/advs.202307216 PMC11267308 38767134

[21] Wang H, Xu M, Zhang T, Pan J, Li C, Pan B, et al. PYCR1 promotes liver cancer cell growth and metastasis by regulating IRS1 expression through lactylation modification. Clinical Transl Med. 2024;14:e70045. 39422696 10.1002/ctm2.70045 PMC11488319

[22] Hou X, Ouyang J, Tang L, Wu P, Deng X, Yan Q, et al. KCNK1 promotes proliferation and metastasis of breast cancer cells by activating lactate dehydrogenase A (LDHA) and up-regulating H3K18 lactylation. Plos Biol. 2024;22:e3002666. 38905316 10.1371/journal.pbio.3002666 PMC11192366

[23] Huang Y, Wang C, Zhou T, Xie F, Liu Z, Xu H, et al. Lumican promotes calcific aortic valve disease through H3 histone lactylation. Eur Heart J. 2024;45:3871–85. 38976370 10.1093/eurheartj/ehae407

[24] Gong F, Zheng X, Xu W, Xie R, Liu W, Pei L, et al. H3K14la drives endothelial dysfunction in sepsis-induced ARDS by promoting SLC40A1/transferrin-mediated ferroptosis. MedComm. 2025;6:e70049. 39822760 10.1002/mco2.70049 PMC11733091

[25] Jiang Z, Xiong N, Yan R, Li S, Liu H, Mao Q, et al. PDHX acetylation facilitates tumor progression by disrupting PDC assembly and activating lactylation-mediated gene expression. Protein Cell. 2025;16:49–63. 39311688 10.1093/procel/pwae052 PMC11700603

[26] Wang H, Xia H, Bai J, Wang Z, Wang Y, Lin J, et al. H4K12 lactylation-regulated NLRP3 is involved in cigarette smoke-accelerated Alzheimer-like pathology through mTOR-regulated autophagy and activation of microglia. J Hazard Mater. 2025;488:137310. 39862777 10.1016/j.jhazmat.2025.137310

[27] Pan R, He L, Zhang J, Liu X, Liao Y, Gao J, et al. Positive feedback regulation of microglial glucose metabolism by histone H4 lysine 12 lactylation in Alzheimer’s disease. Cell Metab. 2022;34:634–48. 35303422 10.1016/j.cmet.2022.02.013

[28] Wang X, Liu X, Xiao R, Fang Y, Zhou F, Gu M, et al. Histone lactylation dynamics: Unlocking the triad of metabolism, epigenetics, and immune regulation in metastatic cascade of pancreatic cancer. Cancer Lett. 2024;598:217117. 39019144 10.1016/j.canlet.2024.217117

[29] Zhao Y, Zhang H, Zhou B, Wan R, Yan Y, He R, et al. The splicing factor SF3B1 is essential for proper alternative splicing and zygotic genome activation in early porcine embryos. Int J Biol Macromol. 2024;282:137401. 39521214 10.1016/j.ijbiomac.2024.137401

[30] Zhang F, Zhou J, Lu P, Zhang X, Yang L, Wu J, et al. Lactylation of histone by BRD4 regulates astrocyte polarization after experimental subarachnoid hemorrhage. J Neuroinflamm. 2024;21:186. 10.1186/s12974-024-03185-6 PMC11290164 39080649

[31] Zhang X, Liu Y, Wang N. Dynamic changes in histone lysine lactylation during meiosis prophase I in mouse spermatogenesis. Proceedings Natl Acad Sci. 2025;122:e1876274174. 10.1073/pnas.2418693122 PMC11848400 39928879

[32] Zhu W, Zeng S, Zhu S, Zhang Z, Zhao R, Qiu Q, et al. Histone H2B lysine lactylation modulates the NF-κB response via KPNA2 during CSFV infection. Int J Biol Macromol. 2025;299:139973. 39826749 10.1016/j.ijbiomac.2025.139973

[33] Wang S, Huang T, Wu Q, Yuan H, Wu X, Yuan F, et al. Lactate reprograms glioblastoma immunity through CBX3-regulated histone lactylation. J Clin Invest. 2024;134:e176851. 39545414 10.1172/JCI176851 PMC11563687

[34] Huang Z, Zhang X, Zhang L, Liu L, Zhang J, Sun Y, et al. STAT5 promotes PD-L1 expression by facilitating histone lactylation to drive immunosuppression in acute myeloid leukemia. Signal Transduct Target Ther. 2023;8:391. 37777506 10.1038/s41392-023-01605-2 PMC10542808

[35] Chen H, Li Y, Li H, Chen X, Fu H, Mao D, et al. NBS1 lactylation is required for efficient DNA repair and chemotherapy resistance. Nature. 2024;631:663–9. 38961290 10.1038/s41586-024-07620-9 PMC11254748

[36] Jin J, Bai L, Wang D, Ding W, Cao Z, Yan P, et al. SIRT3 -dependent delactylation of cyclinE2 prevents hepatocellular carcinoma growth. Embo Rep. 2023;24:e56052. 36896611 10.15252/embr.202256052 PMC10157311

[37] Lu Z, Fang P, Li S, Xia D, Zhang J, Wu X, et al. Lactylation of Histone H3k18 and Egr1 Promotes Endothelial Glycocalyx Degradation in Sepsis-Induced Acute Lung Injury. Adv Sci. 2025;12:2407064. 10.1002/advs.202407064 PMC11831459 39721014

[38] Li J, Shi X, Xu J, Wang K, Hou F, Luan X, et al. Aldehyde dehydrogenase 2 lactylation aggravates mitochondrial dysfunction by disrupting PHB2 mediated mitophagy in acute kidney injury. Adv Sci. 2025;12:2411943. 10.1002/advs.202411943 PMC11848585 39737891

[39] Du S, Zhang X, Jia Y, Peng P, Kong Q, Jiang S, et al. Hepatocyte HSPA12A inhibits macrophage chemotaxis and activation to attenuate liver ischemia/reperfusion injury via suppressing glycolysis-mediated HMGB1 lactylation and secretion of hepatocytes. Theranostics. 2023;13:3856–71. 37441587 10.7150/thno.82607 PMC10334822

[40] Sun L, Wu S, Wang H, Zhang T, Zhang M, Bai X, et al. PDCD6 regulates lactate metabolism to modulate LC3-associated phagocytosis and antibacterial defense. Nat Commun. 2024;15:10157. 39578445 10.1038/s41467-024-54377-w PMC11584876

[41] Gao R, Li Y, Xu Z, Zhang F, Xu J, Hu Y, et al. Mitochondrial pyruvate carrier 1 regulates fatty acid synthase lactylation and mediates treatment of nonalcoholic fatty liver disease. Hepatology. 2023;78:1800–15. 36651176 10.1097/HEP.0000000000000279

[42] Li Q, Zhang F, Wang H, Tong Y, Fu Y, Wu K, et al. NEDD4 lactylation promotes APAP induced liver injury through Caspase11 dependent non-canonical pyroptosis. Int J Biol Sci. 2024;20:1413–35. 38385085 10.7150/ijbs.91284 PMC10878146

[43] Li W, Zhou J, Gu Y, Chen Y, Huang Y, Yang J, et al. Lactylation of RNA m6 A demethylase ALKBH5 promotes innate immune response to DNA herpesviruses and mpox virus. Proceedings Natl Acad Sci. 2024;121:e1885835175. 10.1073/pnas.2409132121 PMC11513906 39413129

[44] Liu B, Tian X, Li L, Zhang R, Wu J, Jiang N, et al. Severe fever with thrombocytopenia syndrome virus induces lactylation of m6A reader protein YTHDF1 to facilitate viral replication. Embo Rep. 2024;25:5599–619. 39496835 10.1038/s44319-024-00310-7 PMC11624280

[45] Tong H, Jiang Z, Song L, Tan K, Yin X, He C, et al. Dual impacts of serine/glycine-free diet in enhancing antitumor immunity and promoting evasion via PD-L1 lactylation. Cell Metab. 2024;36:2493–510. 39577415 10.1016/j.cmet.2024.10.019

[46] Solier S, Müller S, Cañeque T, Versini A, Mansart A, Sindikubwabo F, et al. A druggable copper-signalling pathway that drives inflammation. Nature. 2023;617:386–94. 37100912 10.1038/s41586-023-06017-4 PMC10131557

[47] Liu J, Han X, Zhang T, Tian K, Li Z, Luo F. Reactive oxygen species (ROS) scavenging biomaterials for anti-inflammatory diseases: from mechanism to therapy. J Hematol Oncol. 2023;16:116. 38037103 10.1186/s13045-023-01512-7 PMC10687997

[48] Newton K, Dixit VM, Kayagaki N. Dying cells fan the flames of inflammation. Science. 2021;374:1076–80. 34822265 10.1126/science.abi5934

[49] Morante-Palacios O, Fondelli F, Ballestar E, Martínez-Cáceres EM. Tolerogenic dendritic cells in autoimmunity and inflammatory diseases. Trends Immunol. 2021;42:59–75. 33293219 10.1016/j.it.2020.11.001

[50] Schnell A, Littman DR, Kuchroo VK. TH17 cell heterogeneity and its role in tissue inflammation. Nat Immunol. 2023;24:19–29. 36596896 10.1038/s41590-022-01387-9 PMC10795475

[51] Tschopp CM, Spiegl N, Didichenko S, Lutmann W, Julius P, Virchow JC, et al. Granzyme B, a novel mediator of allergic inflammation: its induction and release in blood basophils and human asthma. Blood. 2006;108:2290–9. 16794249 10.1182/blood-2006-03-010348

[52] Clayton KL, Collins DR, Lengieza J, Ghebremichael M, Dotiwala F, Lieberman J, et al. Resistance of HIV-infected macrophages to CD8+ T lymphocyte–mediated killing drives activation of the immune system. Nat Immunol. 2018;19:475–86. 29670239 10.1038/s41590-018-0085-3 PMC6025741

[53] Meng B, Li Y, Ding Y, Xu X, Wang L, Guo B, et al. Myeloid-derived growth factor inhibits inflammation and alleviates endothelial injury and atherosclerosis in mice. Sci Adv. 2021;7:e6903. 10.1126/sciadv.abe6903 PMC8139583 34020949

[54] Magisetty J, Pendurthi UR, Esmon CT, Rao LVM. EPCR deficiency or function-blocking antibody protects against joint bleeding–induced pathology in hemophilia mice. Blood. 2020;135:2211–23. 32294155 10.1182/blood.2019003824 PMC7316205

[55] Kim J, Seo D, Yoo S, Lee H, Kim J, Yeom JE, et al. Lung-homing nanoliposomes for early intervention in NETosis and inflammation during acute lung injury. Nano Convergence. 2025;12:8. 39894864 10.1186/s40580-025-00475-4 PMC11788270

[56] Toldo S, Abbate A. The role of the NLRP3 inflammasome and pyroptosis in cardiovascular diseases. Nat Rev Cardiol. 2024;21:219–37. 37923829 10.1038/s41569-023-00946-3 PMC11550901

[57] Li Y, Huang H, Liu B, Zhang Y, Pan X, Yu X, et al. Inflammasomes as therapeutic targets in human diseases. Signal Transduct Target Ther. 2021;6:247. 34210954 10.1038/s41392-021-00650-z PMC8249422

[58] Liu J, Zhang X, Chen K, Cheng Y, Liu S, Xia M, et al. CCR7 Chemokine Receptor-Inducible lnc-Dpf3 restrains dendritic cell migration by inhibiting HIF-1α-Mediated Glycolysis. Immunity. 2019;50:600–15. 30824325 10.1016/j.immuni.2019.01.021

[59] Feng T, Zhao X, Gu P, Yang W, Wang C, Guo Q, et al. Adipocyte-derived lactate is a signalling metabolite that potentiates adipose macrophage inflammation via targeting PHD2. Nat Commun. 2022;13:5208. 36064857 10.1038/s41467-022-32871-3 PMC9445001

[60] Souto Carneiro MM, Klika KD, Abreu MT, Meyer AP, Saffrich R, Sandhoff R, et al. Effect of increased lactate dehydrogenase A activity and aerobic glycolysis on the proinflammatory profile of autoimmune CD8+ T cells in rheumatoid arthritis. Arthritis Rheumatol. 2020;72:2050–64. 32602217 10.1002/art.41420

[61] Xu S, Deng K, Lu C, Fu X, Zhu Q, Wan S, et al. Interleukin-6 classic and trans-signaling utilize glucose metabolism reprogramming to achieve anti- or pro-inflammatory effects. Metabolism. 2024;155:155832. 38438106 10.1016/j.metabol.2024.155832

[62] Ye Q, Wang B, Mao J. The pathogenesis and treatment of the ‘Cytokine Storm’ in COVID-19. J Infect. 2020;80:607–13. 32283152 10.1016/j.jinf.2020.03.037 PMC7194613

[63] Fajgenbaum DC, June CH. Cytokine Storm. New Engl J Med. 2020;383:2255–73. 33264547 10.1056/NEJMra2026131 PMC7727315

[64] Triantafyllou E, Woollard KJ, McPhail MJW, Antoniades CG, Possamai LA. The role of monocytes and macrophages in acute and acute-on-chronic liver failure. Front Immunol. 2018;9:2948. 30619308 10.3389/fimmu.2018.02948 PMC6302023

[65] Colombo PC, Ganda A, Lin J, Onat D, Harxhi A, Iyasere JE, et al. Inflammatory activation: cardiac, renal, and cardio-renal interactions in patients with the cardiorenal syndrome. Heart Fail Rev. 2012;17:177–90. 21688186 10.1007/s10741-011-9261-3 PMC3876739

[66] Xu Y, Ma X, Ni W, Zheng L, Lin Z, Lai Y, et al. PKM2-driven lactate overproduction triggers endothelial-to-mesenchymal transition in ischemic flap via mediating TWIST1 lactylation. Adv Sci. 2024;11:2406184. 10.1002/advs.202406184 PMC11653614 39474980

[67] Huang J, Wang X, Li N, Fan W, Li X, Zhou Q, et al. YY1 lactylation aggravates autoimmune uveitis by enhancing microglial functions via inflammatory genes. Adv Sci. 2024;11:2308031. 10.1002/advs.202308031 PMC11109619 38493498

[68] Zhang Y, Gao Y, Wang Y, Jiang Y, Xiang Y, Wang X, et al. RBM25 is required to restrain inflammation via ACLY RNA splicing-dependent metabolism rewiring. Cell Mol Immunol. 2024;21:1231–50. 39251781 10.1038/s41423-024-01212-3 PMC11527992

[69] An S, Yao Y, Hu H, Wu J, Li J, Li L, et al. PDHA1 hyperacetylation-mediated lactate overproduction promotes sepsis-induced acute kidney injury via Fis1 lactylation. Cell Death Dis. 2023;14:457. 37479690 10.1038/s41419-023-05952-4 PMC10362039

[70] Zhao Q, Wang Q, Yao Q, Yang Z, Li W, Cheng X, et al. Nonenzymatic lysine d-lactylation induced by glyoxalase II substrate SLG dampens inflammatory immune responses. Cell Res. 2025;35:97–116. 39757301 10.1038/s41422-024-01060-w PMC11770101

[71] Carroll SL, Pasare C, Barton GM. Control of adaptive immunity by pattern recognition receptors. Immunity. 2024;57:632–48. 38599163 10.1016/j.immuni.2024.03.014 PMC11037560

[72] O’Neill LAJ, Golenbock D, Bowie AG. The history of Toll-like receptors — redefining innate immunity. Nat Rev Immunol. 2013;13:453–60. 23681101 10.1038/nri3446

[73] Palsson-McDermott EM, Curtis AM, Goel G, Lauterbach MAR, Sheedy FJ, Gleeson LE, et al. Pyruvate Kinase M2 Regulates Hif-1α Activity and IL-1β Induction and Is a Critical Determinant of the Warburg Effect in LPS-Activated Macrophages. Cell Metab. 2015;21:65–80. 25565206 10.1016/j.cmet.2014.12.005 PMC5198835

[74] Goretzki A, Lin Y, Zimmermann J, Rainer H, Junker A, Wolfheimer S, et al. Role of Glycolysis and Fatty Acid Synthesis in the Activation and T Cell-Modulating Potential of Dendritic Cells Stimulated with a TLR5-Ligand Allergen Fusion Protein. Int J Mol Sci. 2022;23:12695. 36293550 10.3390/ijms232012695 PMC9604253

[75] Beam JE, Wagner NJ, Lu K, Parsons JB, Fowler VG, Rowe SE, et al. Inflammasome-mediated glucose limitation induces antibiotic tolerance in Staphylococcus aureus. iScience. 2023;26:107942. 37790275 10.1016/j.isci.2023.107942 PMC10543182

[76] Huang W, Zhang J, Miao C, Ying H, Zhang X, Song M, et al. Aflatoxin B1-Induced Testosterone Biosynthesis Disorder via the ROS/AMPK Signaling Pathway in Male Mice. J Agr Food Chem. 2024;72:5955–65. 38451160 10.1021/acs.jafc.3c08769

[77] Yan Q, Zhou J, Gu Y, Huang W, Ruan M, Zhang H, et al. Lactylation of NAT10 promotes N4-acetylcytidine modification on tRNASer-CGA-1-1 to boost oncogenic DNA virus KSHV reactivation. Cell Death Differ. 2024;31:1362–74. 38879723 10.1038/s41418-024-01327-0 PMC11445560

[78] Li H, Liu C, Li R, Zhou L, Ran Y, Yang Q, et al. AARS1 and AARS2 sense l-lactate to regulate cGAS as global lysine lactyltransferases. Nature. 2024;634:1229–37. 39322678 10.1038/s41586-024-07992-y

[79] Matsuzono K, Mashiko T, Koide R, Yoshizumi H, Fujimoto S. Comparison of Prognosis and Cognitive Function of Holistic Neurological Disease: Tochigi Neurological Disease Cohort Study. Journal Alzheimer’s Dis. 2024;98:275–85. 10.3233/JAD-231390 38393916

[80] Wilson L, Stewart W, Dams-O’Connor K, Diaz-Arrastia R, Horton L, Menon DK, et al. The chronic and evolving neurological consequences of traumatic brain injury. Lancet Neurol. 2017;16:813–25. 28920887 10.1016/S1474-4422(17)30279-X PMC9336016

[81] Cacabelos R, Torrellas C, Fernández-Novoa L, Aliev G. Neuroimmune Crosstalk in CNS Disorders: The Histamine Connection. Curr Pharm Des. 2016;22:819–48. 26648474 10.2174/1381612822666151209150954

[82] Sen A, Jette N, Husain M, Sander JW. Epilepsy in older people. Lancet. 2020;395:735–48. 32113502 10.1016/S0140-6736(19)33064-8

[83] Feigin VL, Nichols E, Alam T, Bannick MS, Beghi E, Blake N, et al. Global, regional, and national burden of neurological disorders, 1990–2016: a systematic analysis for the Global Burden of Disease Study 2016. Lancet Neurol. 2019;18:459–80. 30879893 10.1016/S1474-4422(18)30499-X PMC6459001

[84] Feigin VL, Stark BA, Johnson CO, Roth GA, Bisignano C, Abady GG, et al. Global, regional, and national burden of stroke and its risk factors, 1990–2019: a systematic analysis for the Global Burden of Disease Study 2019. Lancet Neurol. 2021;20:795–820. 34487721 10.1016/S1474-4422(21)00252-0 PMC8443449

[85] van Asch CJ, Luitse MJ, Rinkel GJ, van der Tweel I, Algra A, Klijn CJ. Incidence, case fatality, and functional outcome of intracerebral haemorrhage over time, according to age, sex, and ethnic origin: a systematic review and meta-analysis. Lancet Neurol. 2010;9:167–76. 20056489 10.1016/S1474-4422(09)70340-0

[86] Hemphill JC, Greenberg SM, Anderson CS, Becker K, Bendok BR, Cushman M, et al. Guidelines for the Management of Spontaneous Intracerebral Hemorrhage. Stroke. 2015;46:2032–60. 26022637 10.1161/STR.0000000000000069

[87] Langhorne P, Bernhardt J, Kwakkel G. Stroke rehabilitation. Lancet. 2011;377:1693–702. 21571152 10.1016/S0140-6736(11)60325-5

[88] Atri A. The Alzheimer’s Disease Clinical Spectrum. Med Clin N. Am. 2019;103:263–93. 30704681 10.1016/j.mcna.2018.10.009

[89] Bloem BR, Okun MS, Klein C. Parkinson’s disease. Lancet. 2021;397:2284–303. 33848468 10.1016/S0140-6736(21)00218-X

[90] Feldman EL, Goutman SA, Petri S, Mazzini L, Savelieff MG, Shaw PJ, et al. Amyotrophic lateral sclerosis. Lancet. 2022;400:1363–80. 36116464 10.1016/S0140-6736(22)01272-7 PMC10089700

[91] Yuan MM, Peng X, Zeng TY, Wu MLY, Chen Y, Zhang K, et al. The illness experience for people with amyotrophic lateral sclerosis: A qualitative study. J Clin Nurs. 2021;30:1455–63. 33559184 10.1111/jocn.15697 PMC8248064

[92] Mary A, Mancuso R, Heneka MT. Immune Activation in Alzheimer Disease. Annu Rev Immunol. 2024;42:585–613. 38424470 10.1146/annurev-immunol-101921-035222

[93] Boxer AL, Sperling R. Accelerating Alzheimer’s therapeutic development: The past and future of clinical trials. Cell. 2023;186:4757–72. 37848035 10.1016/j.cell.2023.09.023 PMC10625460

[94] Congdon EE, Ji C, Tetlow AM, Jiang Y, Sigurdsson EM. Tau-targeting therapies for Alzheimer disease: current status and future directions. Nat Rev Neurol. 2023;19:715–36. 37875627 10.1038/s41582-023-00883-2 PMC10965012

[95] Zhang X, Liu Y, Rekowski MJ, Wang N. Lactylation of tau in human Alzheimer’s disease brains. Alzheimer’s Dement. 2025;21:e14481. 39740133 10.1002/alz.14481 PMC11851134

[96] An X, He J, Xie P, Li C, Xia M, Guo D, et al. The effect of tau K677 lactylation on ferritinophagy and ferroptosis in Alzheimer’s disease. Free Radic Bio Med. 2024;224:685–706. 39307193 10.1016/j.freeradbiomed.2024.09.021

[97] Tian Q, Li J, Wu B, Pang Y, He W, Xiao Q, et al. APP lysine 612 lactylation ameliorates amyloid pathology and memory decline in Alzheimer’s disease. J Clin Invest. 2025;135:e184656. 39744941 10.1172/JCI184656 PMC11684803

[98] Zhou J, Zhang L, Peng J, Zhang X, Zhang F, Wu Y, et al. Astrocytic LRP1 enables mitochondria transfer to neurons and mitigates brain ischemic stroke by suppressing ARF1 lactylation. Cell Metab. 2024;36:2054–68. 38906140 10.1016/j.cmet.2024.05.016

[99] Weng W, He Z, Ma Z, Huang J, Han Y, Feng Q, et al. Tufm lactylation regulates neuronal apoptosis by modulating mitophagy in traumatic brain injury. Cell Death Differ. 2025;32:530–45. 39496783 10.1038/s41418-024-01408-0 PMC11894137

[100] Yan L, Wang Y, Hu H, Yang D, Wang W, Luo Z, et al. Physical exercise mediates cortical synaptic protein lactylation to improve stress resilience. Cell Metab. 2024;36:2104–17. 39163863 10.1016/j.cmet.2024.07.018

[101] Anand V, Roy SS, Archer SL, Weir EK, Garg SK, Duval S, et al. Trends and Outcomes of Pulmonary Arterial Hypertension–Related Hospitalizations in the United States. Jama Cardiol. 2016;1:1021. 27851838 10.1001/jamacardio.2016.3591 PMC12167389

[102] Conrad N, Molenberghs G, Verbeke G, Zaccardi F, Lawson C, Friday JM, et al. Trends in cardiovascular disease incidence among 22 million people in the UK over 20 years: population based study. BMJ. 2024;385:e78523. 10.1136/bmj-2023-078523 PMC11203392 38925788

[103] McDermott MM, Cella D. Patient Report May Understate Walking Disability in Peripheral Artery Disease. JAMA. 2025;333:1391. 40029646 10.1001/jama.2025.1077

[104] Obel LM, Diederichsen ACP, Kristensen JSS, Gerke O, Larsen KL, Liisberg M, et al. The Nonsyndromic Ascending Thoracic Aorta in a Population-Based Setting. J Am Coll Cardiol. 2025;85:818–31. 39772363 10.1016/j.jacc.2024.10.096

[105] Roth GA, Mensah GA, Johnson CO, Addolorato G, Ammirati E, Baddour LM, et al. Global Burden of Cardiovascular Diseases and Risk Factors, 1990–2019. J Am Coll Cardiol. 2020;76:2982–3021. 33309175 10.1016/j.jacc.2020.11.010 PMC7755038

[106] Hystad P, Larkin A, Rangarajan S, AlHabib KF, Avezum Á, Calik KBT, et al. Associations of outdoor fine particulate air pollution and cardiovascular disease in 157 436 individuals from 21 high-income, middle-income, and low-income countries (PURE): a prospective cohort study. Lancet Planet Health. 2020;4:e235–45. 32559440 10.1016/S2542-5196(20)30103-0 PMC7457447

[107] Sung H, Ferlay J, Siegel RL, Laversanne M, Soerjomataram I, Jemal A, et al. Global Cancer Statistics 2020: GLOBOCAN Estimates of Incidence and Mortality Worldwide for 36 Cancers in 185 Countries. CA: A Cancer J Clinicians. 2021;71:209–49. 10.3322/caac.21660 33538338

[108] Saba L, Cau R, Vergallo R, Kooi ME, Staub D, Faa G, et al. Carotid artery atherosclerosis: mechanisms of instability and clinical implications. Eur Heart J. 2025;46:904–21. 39791527 10.1093/eurheartj/ehae933

[109] Roman M, Miksza J, Lai FY, Sze S, Poppe K, Doughty R, et al. Revascularization in frail patients with acute coronary syndromes: a retrospective longitudinal study. Eur Heart J. 2025;46:535–47. 39548842 10.1093/eurheartj/ehae755 PMC11804245

[110] Ibanez B, James S, Agewall S, Antunes MJ, Bucciarelli-Ducci C, Bueno H, et al. 2017 ESC Guidelines for the management of acute myocardial infarction in patients presenting with ST-segment elevation. Eur Heart J. 2018;39:119–77. 29198432 10.1016/j.rec.2017.11.010

[111] Wang Y, Tan X, Gao H, Yuan H, Hu R, Jia L, et al. Magnitude of Soluble ST2 as a Novel Biomarker for Acute Aortic Dissection. Circulation. 2018;137:259–69. 29146682 10.1161/CIRCULATIONAHA.117.030469

[112] Bak M, Park H, Lee S, Lee N, Ahn M, Ahn JS, et al. The Risk and Reversibility of Osimertinib-Related Cardiotoxicity in a Real-World Population. J Thorac Oncol. 2025;20:167–76. 39395664 10.1016/j.jtho.2024.10.003

[113] Morrow DA, Velazquez EJ, Desai AS, DeVore AD, Lepage S, Park J, et al. Sacubitril/Valsartan in Patients Hospitalized With Decompensated Heart Failure. J Am Coll Cardiol. 2024;83:1123–32. 38508844 10.1016/j.jacc.2024.01.027

[114] Martens P, Burkhoff D, Cowger JA, Jorde UP, Kapur NK, Tang WHW. Emerging Individualized Approaches in the Management of Acute Cardiorenal Syndrome With Renal Assist Devices. JACC: Heart Fail. 2023;11:1289–303. 37676211 10.1016/j.jchf.2023.06.021

[115] Zhang Z, Yao L, Yang J, Wang Z, Du G. PI3K/Akt and HIF‑1 signaling pathway in hypoxia‑ischemia (Review). Mol Med Rep. 2018;18:3547–54. 30106145 10.3892/mmr.2018.9375 PMC6131612

[116] Ramakrishnan S, Anand V, Roy S. Vascular Endothelial Growth Factor Signaling in Hypoxia and Inflammation. J Neuroimmune Pharm. 2014;9:142–60. 10.1007/s11481-014-9531-7 PMC4048289 24610033

[117] Packer M. Hyperuricemia and Gout Reduction by SGLT2 Inhibitors in Diabetes and Heart Failure. J Am Coll Cardiol. 2024;83:371–81. 38199714 10.1016/j.jacc.2023.10.030

[118] Yang S, Li Y, Zhou L, Wang X, Liu L, Wu M. Copper homeostasis and cuproptosis in atherosclerosis: metabolism, mechanisms and potential therapeutic strategies. Cell Death Discov. 2024;10:25. 38218941 10.1038/s41420-023-01796-1 PMC10787750

[119] Tian H, Zhao X, Zhang Y, Xia Z. Abnormalities of glucose and lipid metabolism in myocardial ischemia-reperfusion injury. Biomed Pharmacother. 2023;163:114827. 37141734 10.1016/j.biopha.2023.114827

[120] Eckle T, Hartmann K, Bonney S, Reithel S, Mittelbronn M, Walker LA, et al. Adora2b-elicited Per2 stabilization promotes a HIF-dependent metabolic switch crucial for myocardial adaptation to ischemia. Nat Med. 2012;18:774–82. 22504483 10.1038/nm.2728 PMC3378044

[121] Packer M. Foetal recapitulation of nutrient surplus signalling byO-GlcNAcylation and the failing heart. Eur J Heart Fail. 2023;25:1199–212. 37434410 10.1002/ejhf.2972

[122] Loor G, Schumacker PT. Role of hypoxia-inducible factor in cell survival during myocardial ischemia–reperfusion. Cell Death Differ. 2008;15:686–90. 18259200 10.1038/cdd.2008.13

[123] Fan M, Yang K, Wang X, Chen L, Gill PS, Ha T, et al. Lactate promotes endothelial-to-mesenchymal transition via Snail1 lactylation after myocardial infarction. Sci Adv. 2023;9:c9465. 10.1126/sciadv.adc9465 PMC9897666 36735787

[124] Ouyang F, Li Y, Wang H, Liu X, Tan X, Xie G, et al. Aloe Emodin Alleviates Radiation-Induced Heart Disease via Blocking P4HB Lactylation and Mitigating Kynurenine Metabolic Disruption. Adv Sci. 2024;11:2406026. 10.1002/advs.202406026 PMC11653682 39494721

[125] She H, Hu Y, Zhao G, Du Y, Wu Y, Chen W, et al. Dexmedetomidine Ameliorates Myocardial Ischemia-Reperfusion Injury by Inhibiting MDH2 Lactylation via Regulating Metabolic Reprogramming. Adv Sci. 2024;11:2409499. 10.1002/advs.202409499 PMC11672254 39467114

[126] Wang L, Li D, Yao F, Feng S, Tong C, Rao R, et al. Serpina3k lactylation protects from cardiac ischemia reperfusion injury. Nat Commun. 2025;16:1012. 39856050 10.1038/s41467-024-55589-w PMC11760901

[127] Zhang N, Zhang Y, Xu J, Wang P, Wu B, Lu S, et al. α-myosin heavy chain lactylation maintains sarcomeric structure and function and alleviates the development of heart failure. Cell Res. 2023;33:679–98. 37443257 10.1038/s41422-023-00844-w PMC10474270

[128] Chen L, Zhang M, Yang X, Wang Y, Huang T, Li X, et al. Methyl-CpG-binding 2 K271 lactylation-mediated M2 macrophage polarization inhibits atherosclerosis. Theranostics. 2024;14:4256–77. 39113793 10.7150/thno.94738 PMC11303070

[129] Kiri S, Ryba T. Cancer, metastasis, and the epigenome. Mol Cancer. 2024;23:154. 39095874 10.1186/s12943-024-02069-w PMC11295362

[130] Bhatnagar K, Raju S, Patki N, Motiani RK, Chaudhary S. Targeting mineral metabolism in cancer: Insights into signaling pathways and therapeutic strategies. Semin Cancer Biol. 2025;112:1–19. 40024314 10.1016/j.semcancer.2025.02.011

[131] Chen S, Cao Z, Prettner K, Kuhn M, Yang J, Jiao L, et al. Estimates and Projections of the Global Economic Cost of 29 Cancers in 204 Countries and Territories From 2020 to 2050. Jama Oncol. 2023;9:465. 36821107 10.1001/jamaoncol.2022.7826 PMC9951101

[132] Hofman DA, Prensner JR, van Heesch S. Microproteins in cancer: identification, biological functions, and clinical implications. Trends Genet. 2025;41:146–61. 39379206 10.1016/j.tig.2024.09.002 PMC11794034

[133] Parkin DM, Stjernsward J, Muir CS. Estimates of the worldwide frequency of twelve major cancers. Bull World Health Organ. 1984;62:163–82. 6610488 PMC2536293

[134] Zhao J, Xu L, Sun J, Song M, Wang L, Yuan S, et al. Global trends in incidence, death, burden and risk factors of early-onset cancer from 1990 to 2019. BMJ Oncol. 2023;2:e49. 10.1136/bmjonc-2023-000049 PMC11235000 39886513

[135] Akimoto N, Ugai T, Zhong R, Hamada T, Fujiyoshi K, Giannakis M, et al. Rising incidence of early-onset colorectal cancer — a call to action. Nat Rev Clin Oncol. 2021;18:230–43. 33219329 10.1038/s41571-020-00445-1 PMC7994182

[136] Koritala BSC, Porter KI, Arshad OA, Gajula RP, Mitchell HD, Arman T, et al. Night shift schedule causes circadian dysregulation of DNA repair genes and elevated DNA damage in humans. J Pineal Res. 2021;70:e12726. 33638890 10.1111/jpi.12726 PMC8011353

[137] Lan A, Li H, Shen M, Li D, Shu D, Liu Y, et al. Association of depressive symptoms and sleep disturbances with survival among US adult cancer survivors. Bmc Med. 2024;22:225. 38835034 10.1186/s12916-024-03451-7 PMC11151538

[138] Vishwakarma M, Piddini E. Outcompeting cancer. Nat Rev Cancer. 2020;20:187–98. 31932757 10.1038/s41568-019-0231-8

[139] Zheng J, Wang S, Xia L, Sun Z, Chan KM, Bernards R, et al. Hepatocellular carcinoma: signaling pathways and therapeutic advances. Signal Transduct Target Ther. 2025;10:35. 39915447 10.1038/s41392-024-02075-w PMC11802921

[140] Cui B, Luo H, He B, Liu X, Lv D, Zhang X, et al. Gut dysbiosis conveys psychological stress to activate LRP5/β-catenin pathway promoting cancer stemness. Signal Transduct Target Ther. 2025;10:79. 40038255 10.1038/s41392-025-02159-1 PMC11880501

[141] Wang X, Wang L, Hao Q, Cai M, Wang X, An W. Harnessing glucose metabolism with nanomedicine for cancer treatment. Theranostics. 2024;14:6831–82. 39479443 10.7150/thno.100036 PMC11519798

[142] Yadav D, Yadav A, Bhattacharya S, Dagar A, Kumar V, Rani R. GLUT and HK: Two primary and essential key players in tumor glycolysis. Semin Cancer Biol. 2024;100:17–27. 38494080 10.1016/j.semcancer.2024.03.001

[143] Barron CC, Bilan PJ, Tsakiridis T, Tsiani E. Facilitative glucose transporters: Implications for cancer detection, prognosis and treatment. Metabolism. 2016;65:124–39. 26773935 10.1016/j.metabol.2015.10.007

[144] Li X, Yang Y, Zhang B, Lin X, Fu X, An Y, et al. Lactate metabolism in human health and disease. Signal Transduct Target Ther. 2022;7:305. 36050306 10.1038/s41392-022-01151-3 PMC9434547

[145] Sun P, Ma L, Lu Z. Lactylation: Linking the Warburg effect to DNA damage repair. Cell Metab. 2024;36:1637–9. 39111282 10.1016/j.cmet.2024.06.015

[146] Jiang M, Wang Y, Zhao X, Yu J. From metabolic byproduct to immune modulator: the role of lactate in tumor immune escape. Front Immunol. 2024;15:1492050. 39654883 10.3389/fimmu.2024.1492050 PMC11625744

[147] Li G, Wang D, Zhai Y, Pan C, Zhang J, Wang C, et al. Glycometabolic reprogramming-induced XRCC1 lactylation confers therapeutic resistance in ALDH1A3-overexpressing glioblastoma. Cell Metab. 2024;36:1696–710. 39111285 10.1016/j.cmet.2024.07.011

[148] Ju J, Zhang H, Lin M, Yan Z, An L, Cao Z, et al. The alanyl-tRNA synthetase AARS1 moonlights as a lactyltransferase to promote YAP signaling in gastric cancer. J Clin Invest. 2024;134:e174587. 38512451 10.1172/JCI174587 PMC11093599

[149] Zhao K, Wang X, Zhao D, Lin Q, Zhang Y, Hu Y. lncRNA HITT Inhibits Lactate Production by Repressing PKM2 Oligomerization to Reduce Tumor Growth and Macrophage Polarization. Research. 2022;2022:2022–9854904. 10.34133/2022/9854904 PMC9285634 35909936

[150] Wang JX, Choi SYC, Niu X, Kang N, Xue H, Killam J, et al. Lactic Acid and an Acidic Tumor Microenvironment suppress Anticancer Immunity. Int J Mol Sci. 2020;21:8363. 33171818 10.3390/ijms21218363 PMC7664620

[151] Végran F, Boidot R, Michiels C, Sonveaux P, Feron O. Lactate Influx through the Endothelial Cell Monocarboxylate Transporter MCT1 Supports an NF-κB/IL-8 Pathway that Drives Tumor Angiogenesis. Cancer Res. 2011;71:2550–60. 21300765 10.1158/0008-5472.CAN-10-2828

[152] Huber MA, Azoitei N, Baumann B, Grünert S, Sommer A, Pehamberger H, et al. NF-κB is essential for epithelial-mesenchymal transition and metastasis in a model of breast cancer progression. J Clin Invest. 2004;114:569–81. 15314694 10.1172/JCI21358 PMC503772

[153] Hong H, Han H, Wang L, Cao W, Hu M, Li J, et al. ABCF1-K430-Lactylation promotes HCC malignant progression via transcriptional activation of HIF1 signaling pathway. Cell Death Differ. 2025;32:613–31. 39753865 10.1038/s41418-024-01436-w PMC11982231

[154] Chen B, Deng Y, Hong Y, Fan L, Zhai X, Hu H, et al. Metabolic Recoding of NSUN2-Mediated m5C Modification Promotes the Progression of Colorectal Cancer via the NSUN2/YBX1/m5 C-ENO1 Positive Feedback Loop. Adv Sci. 2024;11:2309840. 10.1002/advs.202309840 PMC11267267 38769664

[155] Zhou Z, Yin X, Sun H, Lu J, Li Y, Fan Y, et al. PTBP1 Lactylation Promotes Glioma Stem Cell Maintenance through PFKFB4-Driven Glycolysis. Cancer Res. 2025;85:739–57. 39570804 10.1158/0008-5472.CAN-24-1412

[156] Lu Y, Zhu J, Zhang Y, Li W, Xiong Y, Fan Y, et al. Lactylation-Driven IGF2BP3-Mediated Serine Metabolism Reprogramming and RNA m6A—Modification Promotes Lenvatinib Resistance in HCC. Adv Sci. 2024;11:2401399. 10.1002/advs.202401399 PMC11633555 39450426

[157] Yu D, Zhong Q, Wang Y, Yin C, Bai M, Zhu J, et al. Lactylation: The metabolic accomplice shaping cancer’s response to radiotherapy and immunotherapy. Ageing Res Rev. 2025;104:102670. 39864560 10.1016/j.arr.2025.102670

[158] Liao J, Chen Z, Chang R, Yuan T, Li G, Zhu C, et al. CENPA functions as a transcriptional regulator to promote hepatocellular carcinoma progression via cooperating with YY1. Int J Biol Sci. 2023;19:5218–32. 37928273 10.7150/ijbs.85656 PMC10620822

[159] Huang H, Wang S, Xia H, Zhao X, Chen K, Jin G, et al. Lactate enhances NMNAT1 lactylation to sustain nuclear NAD+ salvage pathway and promote survival of pancreatic adenocarcinoma cells under glucose-deprived conditions. Cancer Lett. 2024;588:216806. 38467179 10.1016/j.canlet.2024.216806

[160] Chen Y, Wu J, Zhai L, Zhang T, Yin H, Gao H, et al. Metabolic regulation of homologous recombination repair by MRE11 lactylation. Cell. 2024;187:294–311. 38128537 10.1016/j.cell.2023.11.022 PMC11725302

[161] Yang Z, Su W, Zhang Q, Niu L, Feng B, Zhang Y, et al. Lactylation of HDAC1 Confers Resistance to Ferroptosis in Colorectal Cancer. Adv Sci. 2025;12:2408845. 10.1002/advs.202408845 PMC11947995 39888307

[162] Sun L, Zhang Y, Yang B, Sun S, Zhang P, Luo Z, et al. Lactylation of METTL16 promotes cuproptosis via m6A-modification on FDX1 mRNA in gastric cancer. Nat Commun. 2023;14:6523. 37863889 10.1038/s41467-023-42025-8 PMC10589265

[163] Sun H, Li K, Liu C, Yi C. Regulation and functions of non-m6A mRNA modifications. Nat Rev Mol Cell Bio. 2023;24:714–31. 37369853 10.1038/s41580-023-00622-x

[164] Nombela P, Miguel-López B, Blanco S. The role of m6A, m5C and Ψ RNA modifications in cancer: Novel therapeutic opportunities. Mol Cancer. 2021;20:18. 33461542 10.1186/s12943-020-01263-w PMC7812662

[165] Li D, Liu Y, Yang G, He M, Lu L. Recent insights into RNA m5C methylation modification in hepatocellular carcinoma. Biochimica et Biophysica Acta (BBA) - Rev Cancer. 2024;1879:189223. 10.1016/j.bbcan.2024.189223 39577751

[166] Niu K, Chen Z, Li M, Ma G, Deng Y, Zhang J, et al. NSUN2 lactylation drives cancer cell resistance to ferroptosis through enhancing GCLC-dependent glutathione synthesis. Redox Biol. 2025;79:103479. 39742570 10.1016/j.redox.2024.103479 PMC11750563

[167] Yang L, Niu K, Wang J, Shen W, Jiang R, Liu L, et al. Nucleolin lactylation contributes to intrahepatic cholangiocarcinoma pathogenesis via RNA splicing regulation of MADD. J Hepatol. 2024;81:651–66. 38679071 10.1016/j.jhep.2024.04.010

[168] Xie B, Zhang M, Li J, Cui J, Zhang P, Liu F, et al. KAT8-catalyzed lactylation promotes eEF1A2-mediated protein synthesis and colorectal carcinogenesis. Proceedings Natl Acad Sci. 2024;121:e1980839175. 10.1073/pnas.2314128121 PMC10895275 38359291

[169] Meng Q, Zhang Y, Sun H, Yang X, Hao S, Liu B, et al. Human papillomavirus-16 E6 activates the pentose phosphate pathway to promote cervical cancer cell proliferation by inhibiting G6PD lactylation. Redox Biol. 2024;71:103108. 38457903 10.1016/j.redox.2024.103108 PMC10937312

[170] Zong Z, Xie F, Wang S, Wu X, Zhang Z, Yang B, et al. Alanyl-tRNA synthetase, AARS1, is a lactate sensor and lactyltransferase that lactylates p53 and contributes to tumorigenesis. Cell. 2024;187:2375–92. 38653238 10.1016/j.cell.2024.04.002

[171] Feng F, Wu J, Chi Q, Wang S, Liu W, Yang L, et al. Lactylome Analysis Unveils Lactylation-Dependent Mechanisms of Stemness Remodeling in the Liver Cancer Stem Cells. Adv Sci. 2024;11:2405975. 10.1002/advs.202405975 PMC11481176 39099416

[172] Noe JT, Rendon BE, Geller AE, Conroy LR, Morrissey SM, Young LEA, et al. Lactate supports a metabolic-epigenetic link in macrophage polarization. Sci Adv. 2021;7:i8602. 10.1126/sciadv.abi8602 PMC8589316 34767443

[173] Li H, Sun L, Gao P, Hu H. Lactylation in cancer: Current understanding and challenges. Cancer Cell. 2024;42:1803–7. 39393355 10.1016/j.ccell.2024.09.006

[174] Yang Z, Zheng Y, Gao Q. Lysine lactylation in the regulation of tumor biology. Trends Endocrinol Metab. 2024;35:720–31. 38395657 10.1016/j.tem.2024.01.011

[175] Liao Z, Chen B, Yang T, Zhang W, Mei Z. Lactylation modification in cardio-cerebral diseases: A state-of-the-art review. Ageing Res Rev. 2025;104:102631. 39647583 10.1016/j.arr.2024.102631

[176] Zhao L, Qi H, Lv H, Liu W, Zhang R, Yang A. Lactylation in health and disease: physiological or pathological?. Theranostics. 2025;15:1787–821. 39897556 10.7150/thno.105353 PMC11780532

[177] Hu X, Wu X, Xu J, Xu X Lactate-mediated lactylation in human health and diseases: Progress and remaining challenges. J Adv Res. 2024; 10.1016/j.jare.2024.11.010 PMC12536595 39522689

[178] Dichtl S, Lindenthal L, Zeitler L, Behnke K, Schlösser D, Strobl B, et al. Lactate and IL6 define separable paths of inflammatory metabolic adaptation. Sci Adv. 2021;7:g3505. 10.1126/sciadv.abg3505 PMC8221612 34162546

[179] Lin X, Lei Y, Pan M, Hu C, Xie B, Wu W, et al. Augmentation of scleral glycolysis promotes myopia through histone lactylation. Cell Metab. 2024;36:511–25. 38232735 10.1016/j.cmet.2023.12.023

[180] Sun X, He L, Liu H, Thorne RF, Zeng T, Liu L, et al. The diapause-like colorectal cancer cells induced by SMC4 attenuation are characterized by low proliferation and chemotherapy insensitivity. Cell Metab. 2023;35:1563–79. 37543034 10.1016/j.cmet.2023.07.005

[181] Gong T, Wang Q, Loughran PA, Li Y, Scott MJ, Billiar TR, et al. Mechanism of lactic acidemia-promoted pulmonary endothelial cells death in sepsis: role for CIRP-ZBP1-PANoptosis pathway. Military Med Res. 2024;11:71. 10.1186/s40779-024-00574-z PMC11514876 39465383

[182] Wang Y, Li P, Xu Y, Feng L, Fang Y, Song G, et al. Lactate metabolism and histone lactylation in the central nervous system disorders: impacts and molecular mechanisms. J Neuroinflamm. 2024;21:308. 10.1186/s12974-024-03303-4 PMC11605911 39609834

[183] Zhao R, Yi Y, Liu H, Xu J, Chen S, Wu D, et al. RHOF promotes Snail1 lactylation by enhancing PKM2-mediated glycolysis to induce pancreatic cancer cell endothelial–mesenchymal transition. Cancer Metab. 2024;12:32. 39462429 10.1186/s40170-024-00362-2 PMC11515152

[184] Ngoi NYL, Pilié PG, McGrail DJ, Zimmermann M, Schlacher K, Yap TA. Targeting ATR in patients with cancer. Nat Rev Clin Oncol. 2024;21:278–93. 38378898 10.1038/s41571-024-00863-5

[185] Kwok M, Agathanggelou A, Stankovic T. DNA damage response defects in hematologic malignancies: mechanistic insights and therapeutic strategies. Blood. 2024;143:2123–44. 38457665 10.1182/blood.2023019963

[186] Da Costa AABA, Chowdhury D, Shapiro GI, D Andrea AD, Konstantinopoulos PA. Targeting replication stress in cancer therapy. Nat Rev Drug Discov. 2023;22:38–58. 36202931 10.1038/s41573-022-00558-5 PMC11132912

[187] Awwad SW, Serrano-Benitez A, Thomas JC, Gupta V, Jackson SP. Revolutionizing DNA repair research and cancer therapy with CRISPR–Cas screens. Nat Rev Mol Cell Bio. 2023;24:477–94. 36781955 10.1038/s41580-022-00571-x

[188] Cybulla E, Vindigni A. Leveraging the replication stress response to optimize cancer therapy. Nat Rev Cancer. 2023;23:6–24. 36323800 10.1038/s41568-022-00518-6 PMC9789215

[189] Milano L, Gautam A, Caldecott KW. DNA damage and transcription stress. Mol Cell. 2024;84:70–9. 38103560 10.1016/j.molcel.2023.11.014

[190] Zhao BS, Roundtree IA, He C. Post-transcriptional gene regulation by mRNA modifications. Nat Rev Mol Cell Bio. 2017;18:31–42. 27808276 10.1038/nrm.2016.132 PMC5167638

[191] Gilbert WV, Bell TA, Schaening C. Messenger RNA modifications: Form, distribution, and function. Science. 2016;352:1408–12. 27313037 10.1126/science.aad8711 PMC5094196

[192] Chen D, Gu X, Nurzat Y, Xu L, Li X, Wu L, et al. Writers, readers, and erasers RNA modifications and drug resistance in cancer. Mol Cancer. 2024;23:178. 39215288 10.1186/s12943-024-02089-6 PMC11363509

[193] Ganapathy-Kanniappan S, Geschwind JH. Tumor glycolysis as a target for cancer therapy: progress and prospects. Mol Cancer. 2013;12:152. 24298908 10.1186/1476-4598-12-152 PMC4223729

[194] Cheng Q, Shi XL, Li QL, Wang L, Wang Z. Current Advances on Nanomaterials Interfering with Lactate Metabolism for Tumor Therapy. Adv Sci. 2024;11:2305662. 10.1002/advs.202305662 PMC10797484 37941489

[195] García-Cañaveras JC, Chen L, Rabinowitz JD. The Tumor Metabolic Microenvironment: Lessons from Lactate. Cancer Res. 2019;79:3155–62. 31171526 10.1158/0008-5472.CAN-18-3726 PMC6606343

[196] Wen H, Deng H, Li B, Chen J, Zhu J, Zhang X, et al. Mitochondrial diseases: from molecular mechanisms to therapeutic advances. Signal Transduct Target Ther. 2025;10:9. 39788934 10.1038/s41392-024-02044-3 PMC11724432

[197] Decker ST, Funai K. Mitochondrial membrane lipids in the regulation of bioenergetic flux. Cell Metab. 2024;36:1963–78. 39178855 10.1016/j.cmet.2024.07.024 PMC11374467

[198] Czabotar PE, Garcia-Saez AJ. Mechanisms of BCL-2 family proteins in mitochondrial apoptosis. Nat Rev Mol Cell Bio. 2023;24:732–48. 37438560 10.1038/s41580-023-00629-4

[199] Bock FJ, Tait SWG. Mitochondria as multifaceted regulators of cell death. Nat Rev Mol Cell Bio. 2020;21:85–100. 31636403 10.1038/s41580-019-0173-8

[200] De Leo A, Ugolini A, Yu X, Scirocchi F, Scocozza D, Peixoto B, et al. Glucose-driven histone lactylation promotes the immunosuppressive activity of monocyte-derived macrophages in glioblastoma. Immunity. 2024;57:1105–23. 38703775 10.1016/j.immuni.2024.04.006 PMC11114377

[201] Li S, Dong L, Wang K. Current and future perspectives of lysine lactylation in cancer. Trends Cell Biol. 2025;35:190–3. 39837737 10.1016/j.tcb.2024.12.015

[202] de la Cruz-López KG, Castro-Muñoz LJ, Reyes-Hernández DO, García-Carrancá A, Manzo-Merino J. Lactate in the regulation of tumor microenvironment and therapeutic approaches. Front Oncol. 2019;9:1143. 31737570 10.3389/fonc.2019.01143 PMC6839026

[203] Liberti MV, Locasale JW. Histone lactylation: a new role for glucose metabolism. Trends Biochem Sci. 2020;45:179–82. 31901298 10.1016/j.tibs.2019.12.004

[204] Noberini R, Robusti G, Bonaldi T. Mass spectrometry-based characterization of histones in clinical samples: applications, progress, and challenges. FEBS J. 2022;289:1191–213. 33415821 10.1111/febs.15707 PMC9291046

[205] Dai X, Lv X, Thompson EW, Ostrikov KK. Histone lactylation: epigenetic mark of glycolytic switch. Trends Genet. 2022;38:124–7. 34627643 10.1016/j.tig.2021.09.009

[206] Fan H, Yang F, Xiao Z, Luo H, Chen H, Chen Z, et al. Lactylation: novel epigenetic regulatory and therapeutic opportunities. Am J Physiol-Endoc M. 2023;324:E330–8. 10.1152/ajpendo.00159.2022 36856188

